# From Binding-Induced Dynamic Effects in SH3 Structures to Evolutionary Conserved Sectors

**DOI:** 10.1371/journal.pcbi.1004938

**Published:** 2016-05-23

**Authors:** Ana Zafra Ruano, Elisa Cilia, José R. Couceiro, Javier Ruiz Sanz, Joost Schymkowitz, Frederic Rousseau, Irene Luque, Tom Lenaerts

**Affiliations:** 1 Department of Physical Chemistry and Institute of Biotechnology, University of Granada, Campus Fuentenueva s/n, Granada, Spain; 2 MLG, Départment d’Informatique, Université Libre de Bruxelles, Brussels, Belgium; 3 Interuniversity Institute of Bioinformatics in Brussels (IB2), ULB-VUB, La Plaine Campus, Brussels, Belgium; 4 VIB SWITCH Laboratory, Leuven, Belgium; 5 Department of Cellular and Molecular Medicine, KU Leuven, Campus Gasthuisberg O&N1, Leuven, Belgium; 6 AI-lab, Vakgroep Computerwetenschappen, Vrije Universiteit Brussel, Brussels, Belgium; University of Toronto, CANADA

## Abstract

Src Homology 3 domains are ubiquitous small interaction modules known to act as docking sites and regulatory elements in a wide range of proteins. Prior experimental NMR work on the SH3 domain of Src showed that ligand binding induces long-range dynamic changes consistent with an induced fit mechanism. The identification of the residues that participate in this mechanism produces a chart that allows for the exploration of the regulatory role of such domains in the activity of the encompassing protein. Here we show that a computational approach focusing on the changes in side chain dynamics through ligand binding identifies equivalent long-range effects in the Src SH3 domain. Mutation of a subset of the predicted residues elicits long-range effects on the binding energetics, emphasizing the relevance of these positions in the definition of intramolecular cooperative networks of signal transduction in this domain. We find further support for this mechanism through the analysis of seven other publically available SH3 domain structures of which the sequences represent diverse SH3 classes. By comparing the eight predictions, we find that, in addition to a dynamic pathway that is relatively conserved throughout all SH3 domains, there are dynamic aspects specific to each domain and homologous subgroups. Our work shows for the first time from a structural perspective, which transduction mechanisms are common between a subset of closely related and distal SH3 domains, while at the same time highlighting the differences in signal transduction that make each family member unique. These results resolve the missing link between structural predictions of dynamic changes and the domain sectors recently identified for SH3 domains through sequence analysis.

## Author Summary

Small protein domains as Src Homology 3 often act as docking sites and serve as regulatory elements. To understand their role in the regulation of a protein’s activity, one needs to understand how their backbone and sidechain dynamics are affected when binding to peptides. We have therefore computationally analyzed eight different SH3 domain structures, predicting dynamical effects induced by binding through our MCIT approach that has been shown to correlate well with experimental data. We show first that binding the Src SH3 domain triggers a particular cascade of dynamic effects, which are compatible with an induced fit mechanism reported before. We then combined the predictions for the eight SH3 domains into different consensus models, with the aim of analyzing, for the first time from a structural perspective, commonalities and differences in the transduction mechanisms among these SH3 domains. These consensus results are, on one hand, in agreement with the domain sectors recently identified for the entire family of SH3 domains. On the other hand, they reveal also that differences exist between the different subgroups that were studied here, requiring extensive experimental investigations of the importance of these differences for the proteins wherein these SH3 domains can be found.

## Introduction

Accumulating experimental evidence shows that binding events are transduced into long-distance dynamic changes at the residue level [1–9]. Yet differently from the large modifications typically observed for multi-domain proteins, in small protein domains these changes occur mostly at the level of the side-chain dynamics, at the *ps-ns* time-scale [10, 11]. Allosteric effects can hence be induced by entropic changes alone [12, 13].

As these internal domain dynamics may play an essential role in the function of the domain and the encompassing protein [14], the question of how to identify precisely the residues involved in the process has received significant attention. Moreover, as the experimental effort to obtain these insights can be substantial and often focuses on amino acids with specific features [8], predictive approaches provide important instruments to more easily identify the relevant dynamic changes, which can then afterwards be screened experimentally. These predictive methods encompass procedures that aim to infer this information from either multiple sequence alignments (MSA) [15–17] or structural data [18–22]. The structure-based MCIT approach [18], which has been shown to produce highly accurate predictions, outperforming some other structure-based methods [23], identifies the dynamically affected residues by quantifying the changes in conformational coupling between their side-chains. The method provides a tool to explore the similarities and differences between domain structures belonging to the same family, revealing to what extent these homologs transduce binding information in the same manner. As MCIT identifies the residue locations within the structure that are dynamically affected by binding, one can investigate whether those dynamic changes occur always in the same locations for a selection of domain family members and whether specific amino acids can be found in those residue locations. These predictions for different family members can in turn be combined into consensus models that encompass the re-occurring locations and their amino acid composition. Such a model allows one to analyze the evolutionary similarities and differences over all predictions, linking the structural observations to the protein sectors recently identified through MSA-based analysis [15, 16].

**Fig 1:**
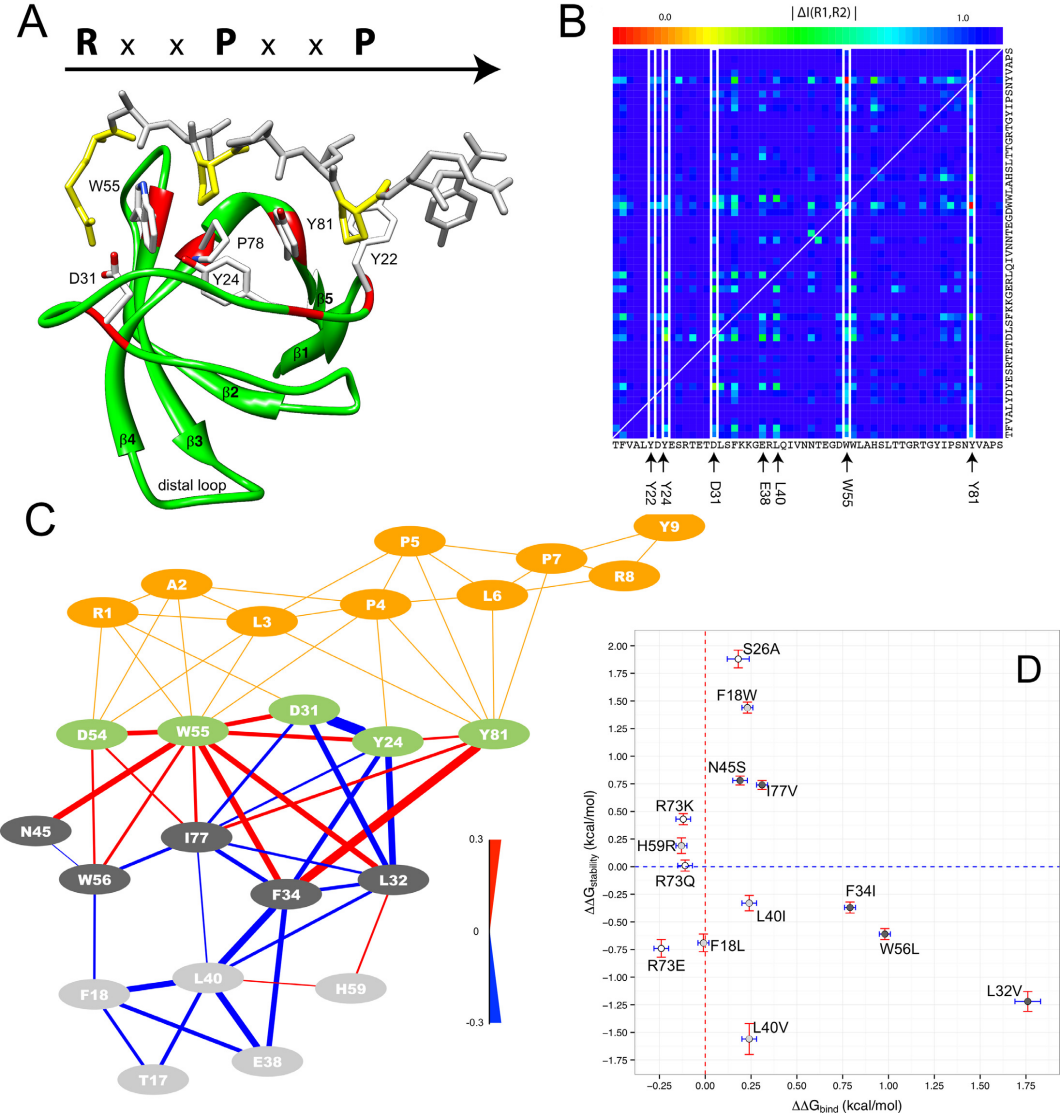
The Src SH3 domain and the matrix of changes in mutual information couplings resulting from our computational analysis. The residue numbering corresponds to that of the MSA shown in Fig 2. A) The structure of the Src SH3 domain (from the NMR ensemble PDB ID:1RLP after energy minimization with Yasara– www.yasara.org) and the most relevant residues of the binding pocket responsible for binding a class I peptide, which forms a polyproline type II (PPII) helix. B) Matrix of the dynamical changes (absolute value of the normalized pair-wise difference between I scores of every residue in the bound and unbound Src SH3 domain). The white boxes highlight the binding pocket residues. C) Set of the 15 residues from the Src SH3 domain predicted as most affected by peptide binding (see also Table 1). The edges represent contacts between residue side-chains observed in at least half of the Src SH3 ensemble structures (see Material and Methods). The color of the edges indicates whether there is a positive (red) or negative (blue) change in mutual information upon the binding event, and their thickness represents the magnitude of this change. Light green nodes are binding-pocket residues and gray nodes are non-binding-pocket residues (dark gray for the first-level subgroup and light gray for the second-level subgroup), while peptide residues are shown in orange. Protein and peptide images were produced with Chimera [41], network images were produced with Cytoscape [42]. D) Effect on the Gibbs energy of folding and binding of selected mutants. White circles represent controls. Residues belonging to the first-level and second-level subgroup are annotated as dark gray and light gray circles respectively. Only mutants in the first-level subgroup have an effect on binding affinity.

To achieve this goal, we apply MCIT here on a collection of SH3 domains for which the necessary structural data is publically available. SH3 domains [24, 25] are small domains, with a typical length of approximately 60 amino acids, which fold into a five-stranded anti-parallel beta-sheet (see Fig 1A). Approximately 300 different instances have been found in the human genome [26], where they participate in a wide variety of cellular processes, ranging from intracellular signaling to immune response. These domains typically recognize proline-rich sequences that contain a core PxxP motif (where x can be almost any other amino acid). In the case of the SH3 domains from the Src family, the PxxP core motif can be flanked by a charged amino acid like R or K, producing class I ([R/K]xxPxxP motif) or clas II (PxxPx[R/K] motif) ligands [27]. Within this sub-family each SH3 domain can bind to both classes, yet favors one of them. Still there are a number of SH3 domains, which have been shown to bind to non-canonical peptide motifs [28, 29]. Given the key role that SH3 domains have been shown to play in different contexts and being very small domains several studies aiming at characterizing their internal dynamics by means of both experimental and computational techniques are available [15, 30–32].

Thermodynamic studies have shown that, in spite of the small size of these domains, the recognition of proline-rich ligands present a significant level of complexity since the binding energetics cannot be explained exclusively in terms of direct interactions within the binding interface. Additional factors such as the presence of interfacial water molecules together with changes in protein dynamics and conformational distribution have been proposed to contribute significantly to the binding energetics [33–39]. In this respect, changes up to 5 kcal·mol^-1^ on the binding enthalpy have been observed associated to conservative mutations more than 10 Å away from the binding site of the Src-SH3 domain. In this context, the identification of the networks of cooperative interactions within the domain, responsible for these long-range effects is of significant importance, especially considering the regulatory role played by these domains in many signal transduction pathways.

**Fig 2:**
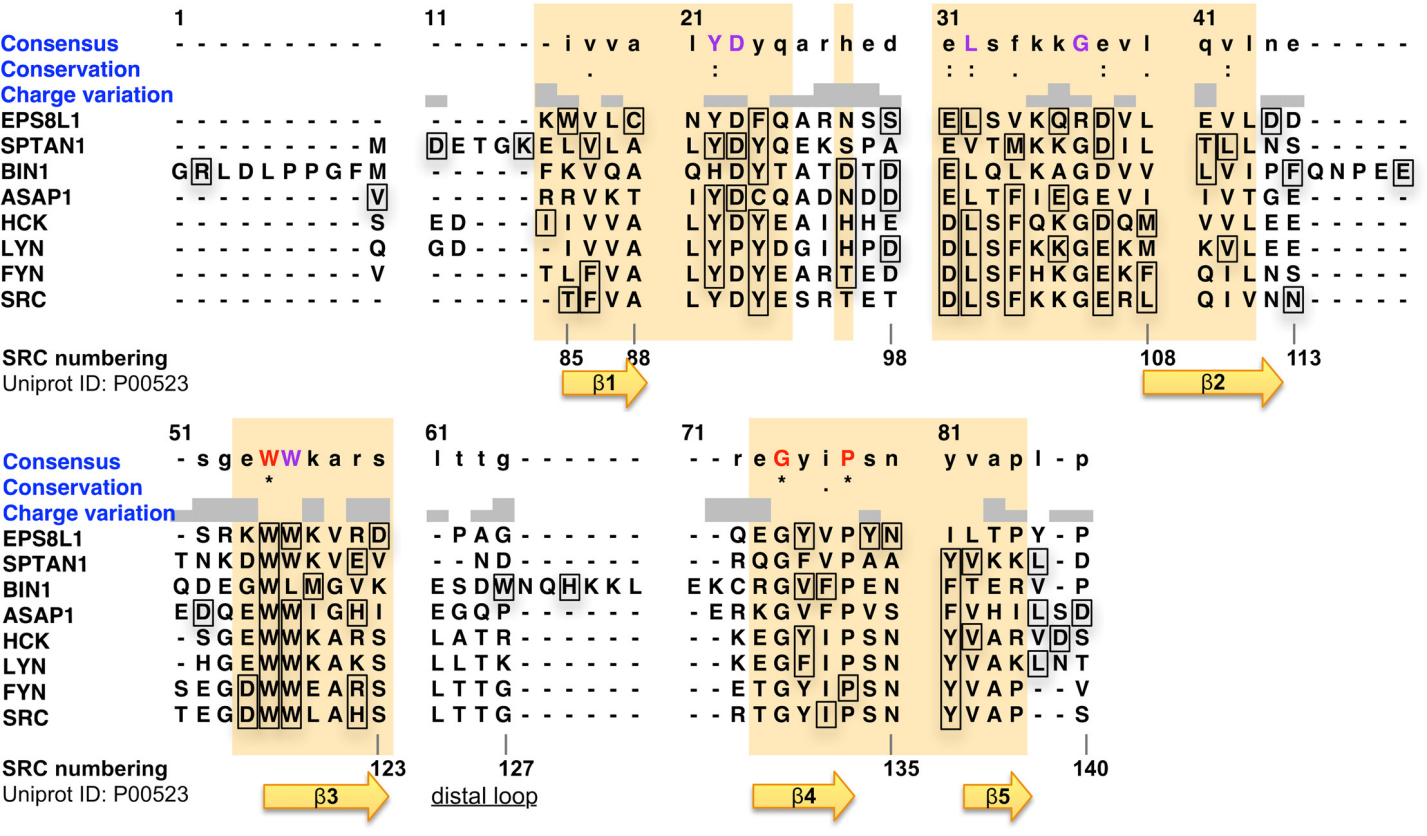
Structural alignment of the SH3 domain variants, with consensus sequence, conservation, and charge variation annotations (illustration provided by Chimera [41]). On top of the alignment, the 15 top-ranking residues are annotated with squares around the amino acid residues. This limit is arbitrary and purely used here for illustration purposes. The regions for which the structural alignment can be considered most reliable are highlighted in orange (see Material and Methods). The Src SH3 numbering from the Uniprot entry P00523 is also reported for reference at the bottom of the alignment, together with the Src SH3 secondary structure annotation.

Our analysis starts, as a point of reference, with the prediction of the residues involved in transducing the binding information through the SH3 domain of Src kinase, which is consistent with previous NMR studies reporting on an induced fit mechanism in Src SH3. Mutation of a subset of the predicted residues elicited long range effects on the binding energetics, underlining the relevance of these positions in the definition of intramolecular cooperative networks of signal transduction in this domain. The same predictions are then performed for seven additional SH3 domains, including three SH3 domains belonging also to the Src-family members and four SH3 domains having low sequence identity (< 35%) with the original Src SH3 domain. Note that throughout the manuscript the numbering resulting from the multiple-sequence alignment (MSA) of all 8 SH3 domains will be used for the residues (see top of Fig 2). We quantify the correlation between the predictions obtained for those SH3 domains and investigate the similarities and differences between the individual Src SH3 domain predictions as well as between different consensus models. Finally, we examine the amino acid conservation in the predicted residue locations. Our analysis shows for the first time from a structural perspective, which transduction mechanisms are common between a subset of closely related and distal SH3 domains, while at the same time highlighting the differences one can observe. Especially these differences may prove to be important in the long run as they could potentially highlight functional characteristics important for each individual SH3 domain within their protein context.

## Results

### Predictions identify long-range dynamic effects in Src SH3

To understand which residues throughout the Src-SH3 structure assist in transducing the peptide-binding event (see in Fig 1A), the MCIT approach is used to determine how the conformational coupling of the residue side-chains differs between the bound and unbound states of the domain (for methodological details see also [23]). For every pair of residues, and in either state, a mutual information (*I*) score is calculated (see Materials and Methods), which measures how strongly the side-chain conformational ensembles of two residues, possibly at distal locations in the structure, are coupled. A high score indicates a strong correlation between the conformational distributions of the two residue sidechains, whereas a low value shows the opposite. Taking the absolute value of the normalized difference between the *I* scores for the bound and unbound states for each pair of residues in the domain, we construct a matrix of dynamical changes (*ΔI*), as is shown in Fig 1B, that visualizes which residue couplings change between both states of the Src SH3 domain. The figure shows that most of the residues do not experience significant changes (dark blue color) in their coupling. As expected, the most significant effects occur among the binding-site residues, although the impact of peptide binding is not homogeneously distributed. Some residues, such as D31, W55, and Y81 experience significant dynamical changes while others (Y22 and Y24) show smaller or even negligible effects. Interestingly, other structural locations and associated amino acids outside the binding pocket experience also significant changes in their conformational coupling to other residues in the structure (see for instance E38 and L40 in Fig 1B).

Using a clustering approach (see Materials and Methods) the collection of residues that experience the largest dynamical changes, which we refer to as the *informative group*, are extracted from the data in the matrix in Fig 1B. The threshold used by the clustering algorithm allows one to determine the expected level of dynamical change between the members incorporated into the cluster, making it possible to add or remove details from the cluster by respectively decreasing or increasing the threshold [18, 23]. Since the threshold determines when certain residue positions become part of the informative group, gradually decreasing it produces a ranking of the residues in terms of how strongly they are dynamically affected by the binding event. The ranking produced for Src SH3 can be observed in the first column of Table 1.

**Table 1:** Summary of the consensus predictions.

Src SH3				Consensus			Consensus			Consensus		
				(Src-related)			(all)			(non Src-related)		
**Src #**	**AA**	**#**	**c. r.**	**AA**	**#**	**c. r.**	**AA**	**#**	**c. r.**	**AA**	**#**	**c. r.**
**118**	**W**	**55†**	**0.99**	**W**	**55†**	**0.99**	**W**	**55†**	**0.99**	**W**	**55†**	**0.98**
**136**	**Y**	**81†**	**0.99**	**y**	**81†**	**0.99**	**y**	**81†**	**0.91**	**e**	**31†**	**0.87**
**102**	F*	**34**	**0.96**	**e**	**31†**	**0.95**	**e**	**31†**	**0.91**	**y**	**81†**	**0.84**
***122***	***H***	***59***	***0*.*95***	**y**	**24†**	**0.91**	**Y**	**22**	**0.86**	**Y**	**22**	**0.83**
***86***	***F***	***18***	***0*.*88***	f*	**34**	**0.91**	**y**	**24†**	**0.86**	W*	**56**	**0.81**
**92**	**Y**	**24†**	**0.88**	**Y**	**22**	**0.90**	W*	**56**	**0.85**	**y**	**24†**	**0.80**
**99**	**D**	**31†**	**0.88**	W*	**56**	**0.89**	f*	**34**	**0.83**	**y**	**76**	**0.79**
***106***	***E***	***38***	***0*.*88***	***e***	***38***	***0*.*87***	***e***	***38***	***0*.*82***	**D**	**23**	**0.78**
***108***	***L***	***40***	***0*.*88***	***l***	***40***	***0*.*83***	**y**	**76**	**0.76**	***e***	***38***	***0*.*77***
**119**	W*	**56**	**0.88**	L*	**32**	**0.82**	**h**	**28**	**0.75**	f*	**34**	**0.76**
**100**	L*	**32**	**0.82**	**h**	**28**	**0.79**	L*	**32**	**0.74**	**q**	**41**	**0.74**
**85**	***T***	***17***	**0.80**	**y**	**76**	**0.73**	***l***	***40***	***0*.*72***	l	85	0.74
**132**	**I**	**77**	**0.79**	***r***	***59***	***0*.*69***	**s**	**60**	**0.67**	**s**	**60**	**0.72**
113	N	45	0.77	**s**	**79**	**0.68**	**D**	**23**	**0.67**	d	30	0.70
**117**	**D**	**54†**	**0.75**	**e**	**54†**	**0.66**	***r***	***59***	***0*.*65***	**h**	**28**	**0.70**
**134**	**S**	**79**	**0.73**	**P**	**78**	**0.64**	e	45	0.64	e	45	0.68
**133**	**P**	**78**	**0.71**	**s**	**60**	**0.62**	***i***	***17***	***0*.*62***	L*	**32**	**0.66**
94	S	26	0.70	**e**	**74**	**0.62**	d	30	0.61	***i***	***17***	***0*.*64***
**91**	**D**	**23**	**0.68**	e	45	0.61	**v**	**42**	**0.59**	***r***	***59***	***0*.*62***
**90**	**Y**	**22**	**0.66**	**s**	**33**	**0.61**	**q**	**41**	**0.58**	***l***	***40***	***0*.*61***
125	T	62	0.64	***i***	***17***	***0*.*60***	**s**	**79**	**0.56**	s	52	0.61
**96**	**T**	**28**	**0.63**	**v**	**42**	**0.58**	**P**	**78**	**0.56**	**v**	**42**	**0.59**
**123**	**S**	**60**	**0.61**	***v***	***18***	***0*.*58***	**i**	**77**	**0.56**	**v**	**82**	**0.59**
**129**	**T**	**74**	**0.59**	**D**	**23**	**0.56**	**k**	**36**	**0.56**	**k**	**36**	**0.58**
**101**	**S**	**33**	**0.57**	**i**	**77**	**0.55**	**e**	**74**	**0.55**	**i**	**77**	**0.57**
114	T	51	0.55	**n**	**80**	**0.55**	s	52	0.55	**-**	**16**	**0.53**
126	T	63	0.54	**k**	**36**	**0.53**	**s**	**33**	**0.53**	**a**	**83**	**0.53**
**135**	**N**	**80**	**0.52**	**k**	**57**	**0.52**	**v**	**82**	**0.52**	**p**	**84**	**0.51**
**137**	**V**	**82**	**0.50**	d	30	0.52	l	85	0.52	**e**	**74**	**0.49**
**131**	**Y**	**76**	**0.48**	**p**	**84**	**0.50**	***v***	***18***	***0*.*52***	**P**	**78**	**0.48**
**109**	**Q**	**41**	**0.46**	s	52	0.49	**p**	**84**	**0.51**	g	64	0.47
**120**	**L**	**57**	**0.45**	t	63	0.48	**n**	**80**	**0.48**	***v***	***18***	***0*.*46***
**107**	**R**	**39**	**0.42**	**v**	**82**	**0.46**	**-**	**16**	**0.45**	**s**	**33**	**0.45**
**110**	**I**	**42**	**0.42**	**q**	**41**	**0.42**	**k**	**57**	**0.45**	**l**	**21**	**0.45**
**87**	**V**	**19**	**0.39**	**v**	**39**	**0.40**	**e**	**54†**	**0.44**	**s**	**79**	**0.45**
124	L	61	0.38	**l**	**43**	**0.39**	t	63	0.41	**v**	**19**	**0.44**
**104**	**K**	**36**	**0.36**	t	62	0.39	**v**	**39**	**0.39**	p	87	0.43
**103**	**K**	**35**	**0.34**	r	27	0.37	p	87	0.38	n	80	0.41
98	T	30	0.31	n	44	0.36	**v**	**19**	**0.37**	e	29	0.40
**111**	**V**	**43**	**0.31**	**q**	**25**	**0.35**	r	27	0.35	**k**	**57**	**0.38**
115	E	52	0.29	l	61	0.35	**l**	**43**	**0.35**	-	51	0.38
140	S	87	0.27	p	87	0.33	g	64	0.35	**v**	**39**	**0.38**
112	N	44	0.23	-	10	0.32	-	10	0.35	-	10	0.37
128	R	73	0.23	**v**	**19**	**0.30**	n	44	0.34	t	63	0.35
**139**	**P**	**84**	**0.23**	**k**	**35**	**0.27**	**q**	**25**	**0.33**	r	73	0.35
**89**	**L**	**21**	**0.19**	e	29	0.24	**l**	**21**	**0.33**	r	27	0.34
95	R	27	0.19	r	73	0.24	e	29	0.32	n	44	0.33
**93**	**E**	**25**	**0.16**	g	64	0.23	r	73	0.29	**q**	**25**	**0.31**
97	E	29	0.14	**l**	**21**	**0.21**	l	61	0.29	**l**	**43**	**0.31**
**105**	**G**	**37**	**0.07**	**G**	**37**	**0.08**	t	62	0.26	**k**	**35**	**0.21**
116	G	53	0.07	g	53	0.08	**k**	**35**	**0.24**	**e**	**54†**	**0.21**
127	G	64	0.07	**G**	**75**	**0.08**	g	53	0.14	g	53	0.20
**130**	**G**	**75**	**0.07**				**G**	**37**	**0.10**	**a**	**58**	**0.15**
							**G**	**75**	**0.07**	t	62	0.14
										G	37	0.13
										G	75	0.06

Predictions derived for the single Src SH3 domain (column 1), the consensus ranking of the four SH3 domains belonging to the Src kinases family (column 2), the consensus ranking of all the eight SH3 domain variants (column 3), and the consensus ranking of the four SH3 domain variants not belonging to the Src kinases family (column 4). Residues are numbered according to their position in the MSA of Fig 2 (see ‘#’ columns) and consensus ranks (c.r. columns) and consensus amino acids (‘AA’ columns) are provided. The initial ‘Src #’ column provides also the Src SH3 numbering relative to the full kinase (Uniprot ID: P00523. Bold rows correspond to the regions where the structural alignment is considered more reliable (see orange regions in Fig 2); therefore also the consensus rank computation is for those positions more reliable. Residues annotated with † are binding-site residues (see also Fig 1C). The remaining residues belonging to the Src top 15 ranked residues are either italic or underlined. The residues belonging to the first-level subgroup of Src SH3 are underlined and an asterisk was added to the residues that experimentally affected the affinity of the domain for the peptide after mutation. The residues belonging to the second-level subgroup (including the histidine in position 59) are shown in italic. Positions that in the consensus ranking contain more than one fourth of missing information due to the presence of alanine residues or gaps in the structural alignment of Fig 2 are not reported in the above table.

Fig 1C shows the 15 most affected residue locations of Src SH3 (see also Table 1). Within this informative group, three different structural subsets can be identified: A) Binding-site residues Y24, D31, W55, and Y81. These highly conserved amino acids are known to be part of the three binding cavities on the surface of most SH3 domains [24, 40]. D31 has also been described to play an important role in determining the binding specificity in Src. Even though D54 is not part of the conserved hydrophobic pockets, it is known to make hydrophobic contacts with the first proline residue in the PxxP core motif and the preceding leucine [24]. Consequently, it could be considered as part of the binding-pocket residues, in direct contact with the peptide ligand; B) residues F18, L32, F34, L40, W56, and I77 in the core of the SH3 domain structure [24, 40]; and C) exposed residues T17, E38, N45, and H59, which are located up to approximately 20 Å away from the peptide. All residues in groups A, B and C have significant values for their *ΔI* scores with all the members of the informative group.

### An induced fit mechanism explains the Src SH3 predictions

In order to further clarify the relationships between the different identified residues, we derived the potential physical interactions between them using the structural data available in the NMR ensembles (Fig 1C). Each edge in this network corresponds to a frequently occurring contact between the residues, where frequently means that in more than 50% of the structures in the ensemble the two residues have at least one pair of atoms at a distance less than 5 Å. By placing the residues in their relative structural locations, the local information exchange between the informative residues becomes visible. Next to the binding-pocket residues (including D54) the network contains a group of residues that directly interact with the binding pocket residues, which we will refer to as the *first-level subgroup* (L32, F34, N45, W56, and I77), and a group of residues that interacts with the residues in the first-level subgroup, which we will refer to as the *second-level subgroup* (T17, F18, E38, L40, and H59). One can additionally observe in Fig 1C that between the binding pocket residues and the first-level subgroup, the conformational couplings become tighter (red edges) upon binding, whereas between the first- and the second-level subgroup the conformation couplings become more flexible (blue edges). This result seems to indicate a switch in the conformational couplings when the Src SH3 domain binds the peptide RALPPLPRY [43]: Prior to binding, residues in the first-level subgroup are conformationally coupled to residues in the second-level subgroup. Peptide binding relaxes this coupling, tightening, in turn, the coupling between the side-chains in the first-level subgroup and those in the binding site. H59 differs from the other second-level subgroup members, as upon binding its conformation becomes more coupled to some of the residues in the second-level subgroup, but these increases in coupling are weak in comparison to those observed between the binding-site residues and those of the first-level subgroup.

This kind of dynamic reorganization of the intradomain interactions upon peptide binding has been previously proposed as one of the underlying factors responsible for the thermodynamic signature of polyproline recognition by SH3 domains [33–35] (driven by strongly favorable binding enthalpies contrary to what could be expected for a mostly hydrophobic interaction [37, 39]). Considering the 15 residues in the informative group in relation to the binding-induced effects on H-bond lengths discussed in [33], we see that our top ranked residues experience H-bond change directly or are in the direct proximity of a residue that experiences an H-bond length change, which is visualized in S1 Table. Most of the non-binding-site residues in Fig 1C, except E38 and N45 (F34 is not listed as the data is missing in [33]), are part of the H-bond networks, regrouping correlated H-bond changes, identified in that work. Nicholson and colleagues argue that these changes in H-bond length can be rationalized by an induced fit mechanism, where binding the RALPPLPRY peptide introduces strains on the H-bond lengths located in proximity as well as at a distance from the binding site. Our predictions corroborate nicely this induced fit mechanism. S2 Table quantifies this observation through a precision and recall analysis for different rank thresholds (10 to 40) applied to the Src SH3 predictions and relative to the residues that experience H-bond changes in [33]. For the threshold 15 for instance, MCIT identifies with 100% precision 50% of the residues whose H-bonds are affected by binding. Increasing this threshold includes more and more affected residues for decreasing levels of precision. Nonetheless, even for a threshold of 25, 90% precision is still obtained for 62% of the affected residues. In addition, the blue lines in Fig 1C between for instance F18-L40 and W56-I77 are supported by the lengthening the H-bonds [33]. These bond length changes seem to affect the side-chain couplings we observe for those residues as well as for the residues in their immediate vicinity (see Fig 1C). Together our results indicate that the coupling changes identified by the MCIT approach provide a good indication of residues that experience binding effects on their H-bonds, seemingly indicating that our predictions serve as a proxy for the changes in the H-bonds.

### Mutation of residues in the first-level subgroup elicits long-range effects on the binding energetics

The prediction results show now that the residues at different distances experience changes due to binding and that an induced fit mechanism explains these results. What remains unclear is whether the residues in Fig 1C have the same role to play in the binding event itself. In other words, do they affect the binding affinity of the RALPPLPRY peptide in the same manner? As little to no data is available to answer this question, we selected several conservative mutations at positions in those subgroups, which were designed, using FoldX [44], to avoid strong perturbations on protein stability. Known mutations in other SH3 domains were preferred, see for instance [45]. We excluded binding-pocket residues and D54 from the mutagenesis study, as it is clear that mutations at these positions will directly affect the binding energetics. Other residues outside the network visualized in Fig 1C were selected as controls (i.e. S26 and R73).

Differential Scanning Calorimetry (DSC) was used to evaluate the effect of the mutation on the conformational equilibrium and stability of the protein. Isothermal Titration Calorimetry (ITC) measured the binding energetics of all mutants to the RLP2 ligand at 25°C. The results for the 15 mutants studied are summarized in Figs 1D, S1 and S2, as well as S3 and S4 Tables.

As predicted, all designed mutants were found to be fully folded and stable at physiological temperatures, with the exception of E38F, which could not be expressed, in agreement with the structural relevance of this position previously described in [46]. A clearly distinct behavior is observed for the first and second level mutants, which may be directly associated to the difference in response to binding as visualized by red and blue edges in Fig 1C.

Most mutations result in changes in stability below 1 kcal·mol^-1^. Even though somewhat bigger effects are observed for the L32V mutant, which establishes numerous stabilizing contacts at the base of the RT-loop, the stronger impact on structural stability is associated to mutants of second level residues (L40V and F18W), implicated in packing interactions within the beta-barrel structure. In agreement with previous reports [47], the S26A mutation, chosen as control, has a significantly increased stability with respect to the wild-type. Most interestingly, significant effects on the binding energetics were observed associated to specific positions (L32V, F34I and W56L) within the first level subgroup, resulting in a 4 to 20 times reduction on binding affinity. This is a remarkable result, considering that these positions are more than 10 Å away from the binding site. In all cases, the effect on binding affinity is mostly associated to changes in the enthalpic contributions, in good agreement with the fact that, according to our predictions, binding of the ligand results in a reorganization of the intra-domain interactions, decoupling the first and second level subgroup and tightening the interactions between the first level subgroup and the binding site. The fact that only a subset of residues elicit effects on binding affinity is also in agreement with previous reports indicating well specified pathways for the transmission of cooperative interactions from the binding site to distant regions in the molecule [31] and the fact that low-stability regions are more efficient in the transmission of cooperative interactions [48, 49]. In this respect, it is interesting to point out that they are located within the highly flexible–RT (L32V & F34I) and–nSrc loops (W56L).

To establish the relevance of the residues in the informative group for the regulation of the Src protein, an extensive mutational study of all residues shown in Fig 1C is required as well as an analysis of the affinity effects of those mutants on binding the intra-molecular linker connecting the SH2 and Kinase domains, which is beyond the scope of this article. Initial data gathered for L32V, F34I and W56L (see S3 Fig and S5 Table) reveal that they may also play a role in the regulatory behavior of the Src protein, motivating further experimental analysis.

### Dynamic changes within SH3 domains correlate with homology

To assess the robustness of our Src SH3 results, the MCIT approach was applied to seven other SH3 domains, for which both the NMR ensembles in ligand-free and ligand-bound states are publically available in the PDB database [50]. This set of homologs includes the SH3 domains of the human Src-family kinases Fyn, Lyn, and Hck (see Material and Methods). Additionally, four other SH3 variants, which have a larger evolutionary distance from Src SH3 (see phylogenetic tree in S4 Fig) and for which the NMR ensembles are also available, were analyzed (see Fig 2 and Material and Methods). In Fig 2 we annotated on the structural alignment (SA) of all SH3 domains, the top-15 of the predicted residues for each SH3 domain (the threshold of 15 residues was arbitrarily selected for illustration purposes, see also Table 1). The more reliably aligned regions have been identified through an additional verification of the structural alignment, highlighting them as orange blocks in Fig 2 (see also Material and Methods). The 42 residues belonging to these well-aligned regions, i.e. about 67% of the alignment, provide the basis for the subsequent comparisons between the results obtained for each SH3 domain.

One can observe in Fig 2 that a substantial number of the most highly ranked positions are consistently identified in those orange regions. In some cases (e.g. D31 and Y81) changes in residue type do not affect the outcome of the prediction. In other cases even when the residue type is conserved the position is only relevant for a subset of the SH3 domains (e.g. columns 78 or 80 in the SA). Over all the 120 top-ranking residues identified in the eight SH3 structures (annotated by squares in Fig 2), 100 residues (5 out of 6) are in the well-aligned orange regions shown in Fig 2, amounting to the 30% of residues in these regions. The remaining 20 predicted residues are located outside those orange regions, corresponding to only 12% of the residues in those other regions.

**Fig 3:**
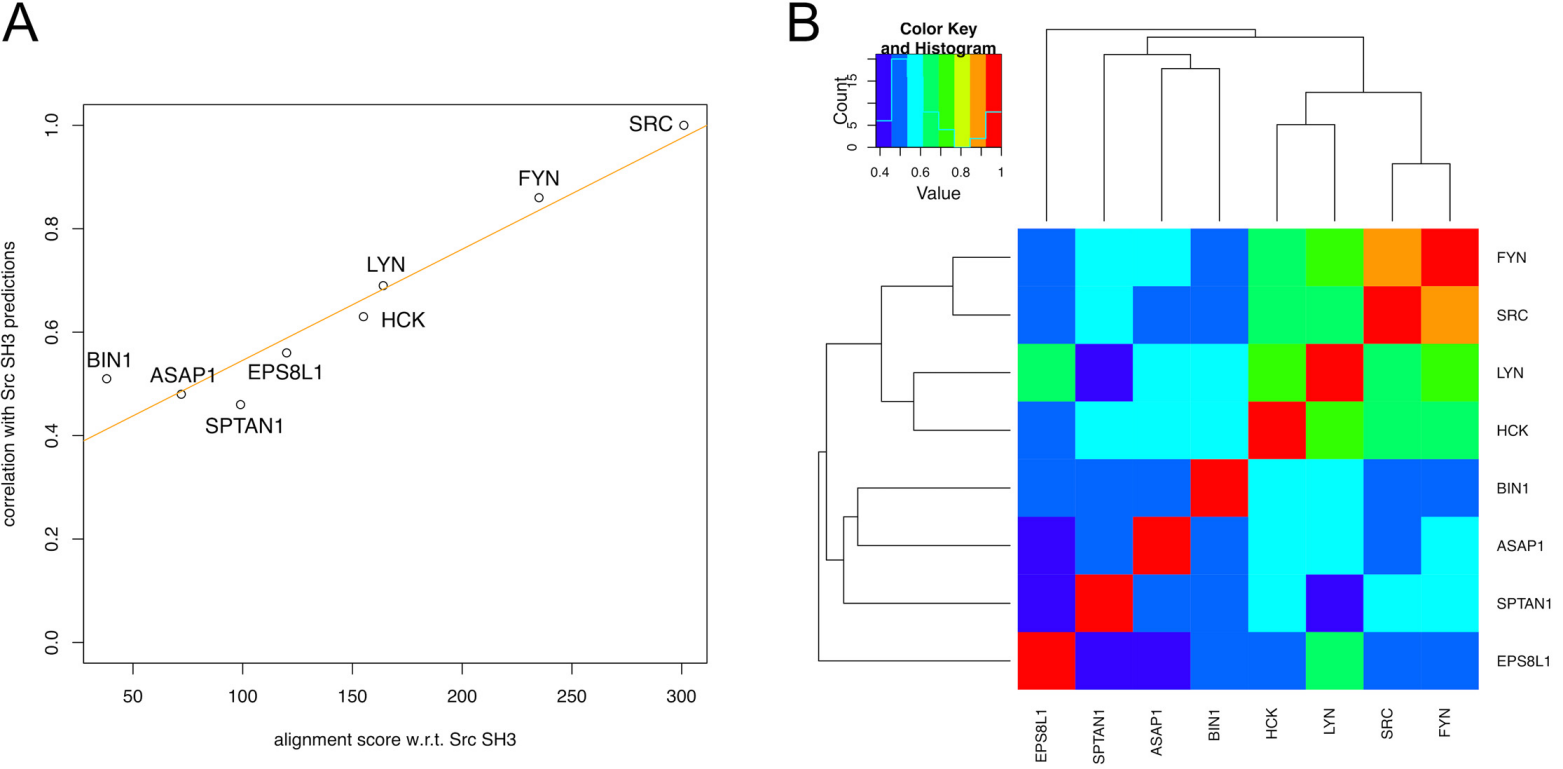
Analysis aimed at showing the degree of conservation of the predictions across the different SH3 variants and its relationship with respect to evolutionary events. A) Scatter plot showing the linear correlation between the alignment scores of each SH3 domain with Src SH3 (x-axis) and the Pearson’s correlation between the rankings produced for each SH3 domain and Src SH3 (y-axis). The regression line is displayed in orange. The Pearson’s correlation between the x- and y-axes values is equal to 0.96. B) Heat map showing the pairwise correlation (Pearson’s correlation coefficients) between the residue rankings obtained for the different SH3 variants (see Materials and Methods). Complete-linkage hierarchical clustering is applied to the matrix of pairwise correlations and the resulting dendrogram is shown on top together with a legend explaining the color-coding.

Using the full rankings for each SH3 domains (like the one provided in Table 1 for Src) the pairwise correlation between the residue rankings was determined to grasp in a quantitative manner the similarity among all the SH3 MCIT-derived rankings (see Material and Methods). First, we compared the result of the pairwise correlation with the Src SH3 domain ranking to the corresponding pairwise sequence alignment score, using the structural alignment in Fig 2 as a basis for the alignment score. As illustrated in Fig 3A, there exists a clear linear relationship between the alignment score and the degree of ranking similarity (Pearson’s correlation equal to 0.96 and p-value of about 10^−4^), indicating that the more an SH3 domain is close in similarity and evolutionary distance (see also the phylogenetic tree in S4 Fig) to the Src SH3, the more the locations predicted to experience dynamic changes of the MCIT method are correlated. This relationship is further confirmed upon analysis of the correlation between all pairs of SH3 domains, shown in Fig 3B, which reproduces the evolutionary relationships. The dendrogram resulting from the comparison clearly highlights the separation between the Src-related SH3 variants, always showing a pairwise correlation above 0.6, and the other four more distant SH3 variants. It is important to stress that this correlation is purely determined by the identified locations not the nature (or identity) of the amino acid found in that location (see Material and Methods).

### Consensus analysis confirms induced fit mechanism

To determine which residues are consistently ranked at the top in all SH3 domains we combined the rankings of each predicted SH3 domain linearly, into consensus rankings or consensus models, based on the alignment in Fig 2 (see Material and Methods). Three consensus models are produced: i) a model built from the sub-group of the four SH3 domains belonging to the Src-family of kinases, ii) a model built from the complete set of eight SH3 domains, and iii) a model built from the sub-group of four SH3 domains not belonging to the Src-family kinases. Table 1 provides detailed information for the ranking of each residue in those three consensus models.

**Fig 4:**
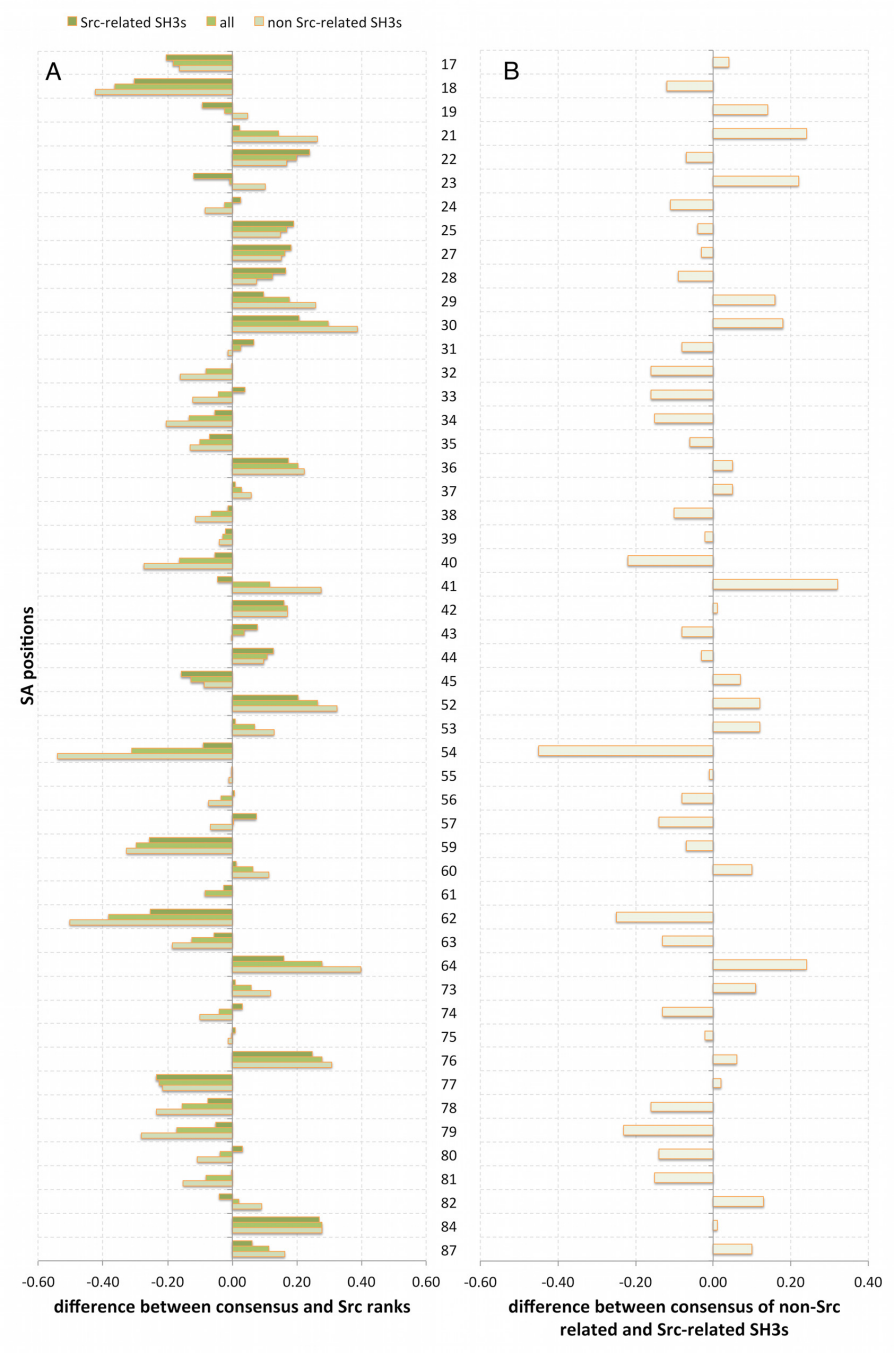
Differences between individual predictions and consensus models. A) Shows the difference in rank between the different consensus models and those obtained for Src SH3 alone. The shading in this bar diagram correlates with the distance from Src (i.e. the lighter the bar the further from Src). B) highlights the ranking differences between the consensus model for the four non-Src-like SH3 domains and the consensus model for the four Src-like SH3 domains. Ranks listed in Table 1 were normalized between 0 and 1, resulting in maximum differences between -1 and 1 in this figure.

To get a better view of the similarities and differences between the models and the ranking obtained for Src SH3 or among the consensus models themselves, we constructed Fig 4: Fig 4A, on the one hand, shows how the specific effects predicted for Src SH3 differ from the three consensus models (the lighter the color of the bar the larger the sequence difference with Src SH3). Negative values indicate that a residue has greater importance in Src than in the consensus models while a positive value indicates the opposite. Both signs can be observed in Fig 4A, revealing that Src SH3 itself has specific dynamic properties which cannot be found in the other SH3 domains. Logically the differences with the consensus model produced for the non-Src related SH3 domains are the greatest, yet in a few cases (e.g. in positions 22, 25 and 28) one can observe the opposite. Notwithstanding those differences one can see in Table 1 that the top ranked residues for Src SH3 maintain their ranking. Fig 4B, on the other hand, shows that both small and larger differences (both negative and positive) occur between the two consensus models for Src-related and non-Src related dynamics, highlighting the fact that differences may be observed within the SH3 family.

Dissecting the information in Fig 4 in more detail one can perceive a number of things. First, one can observe that most binding-site residues (positions 24, 31, 55 and 81) that were already at the top of the ranking change their scores only slightly. Binding-site residues that were missing at the top improve their ranking due to the information provided by the other SH3 domains (see for instance the tyrosine in position 22 and 76 of Src SH3). The most prominent effect can be observed for the residue in position 54, which is highly ranked in all Src-related SH3 domains but experiences a drastic drop (see Fig 4B) in the non-Src-related SH3 domains, and as a consequence also in the consensus model containing all SH3 domains. This drop makes sense when considering the amino-acid composition of the associated peptides and their interaction with the SH3 binding pockets (see Materials and Methods). Also the first-level and second-level subgroup residue rankings remain relatively consistent (see Table 1): N45 for instance, which is not part of a well-aligned (orange) regions in Fig 2, remains important in all the SH3 domains we consider here. Strongest changes are perceived for residues in the second-level subgroup (see position 18, 40, 59 in Fig 4A), except for the residue in position 77, which seems to loose importance in non-Src-related SH3 domains. Note, that other residues, not directly part of the arbitrarily defined informative group, as for instance residues located in position 62 and 64 (which are exactly in the distal loop), also experience drastic effects in Fig 4A, indicating that between SH3 domains, specific residue features may be relevant.

It is interesting to examine the consensus results in Table 1 and Fig 4 also in light of the information provided by the H-bond analysis of Nicholson and colleagues [33] (see S1 Table). Overall the observations made for Src SH3 are consistent between domains: The majority of residues highly ranked in our predictions are also in most cases part of the residue pairs that experience changes in their H-bonds in Src SH3. Moreover, other residues like those in positions 41, 42, 60 and 76, improve their rankings revealing that their role in the induced fit mechanism may be a general property of all SH3 domains studied here. This immediately begs the question whether the amino acids in those locations are also conserved, or at least more conserved than those not highly ranked by the MCIT approach.

### Predicted positions are more conserved than non-predicted positions

Using a conservation analysis performed on the entire SH3 domain family found in PFAM (see Material and Methods), we examine whether the highly ranked residue locations identified for Src SH3 and the consensus models also contain highly conserved amino acids.

**Fig 5:**
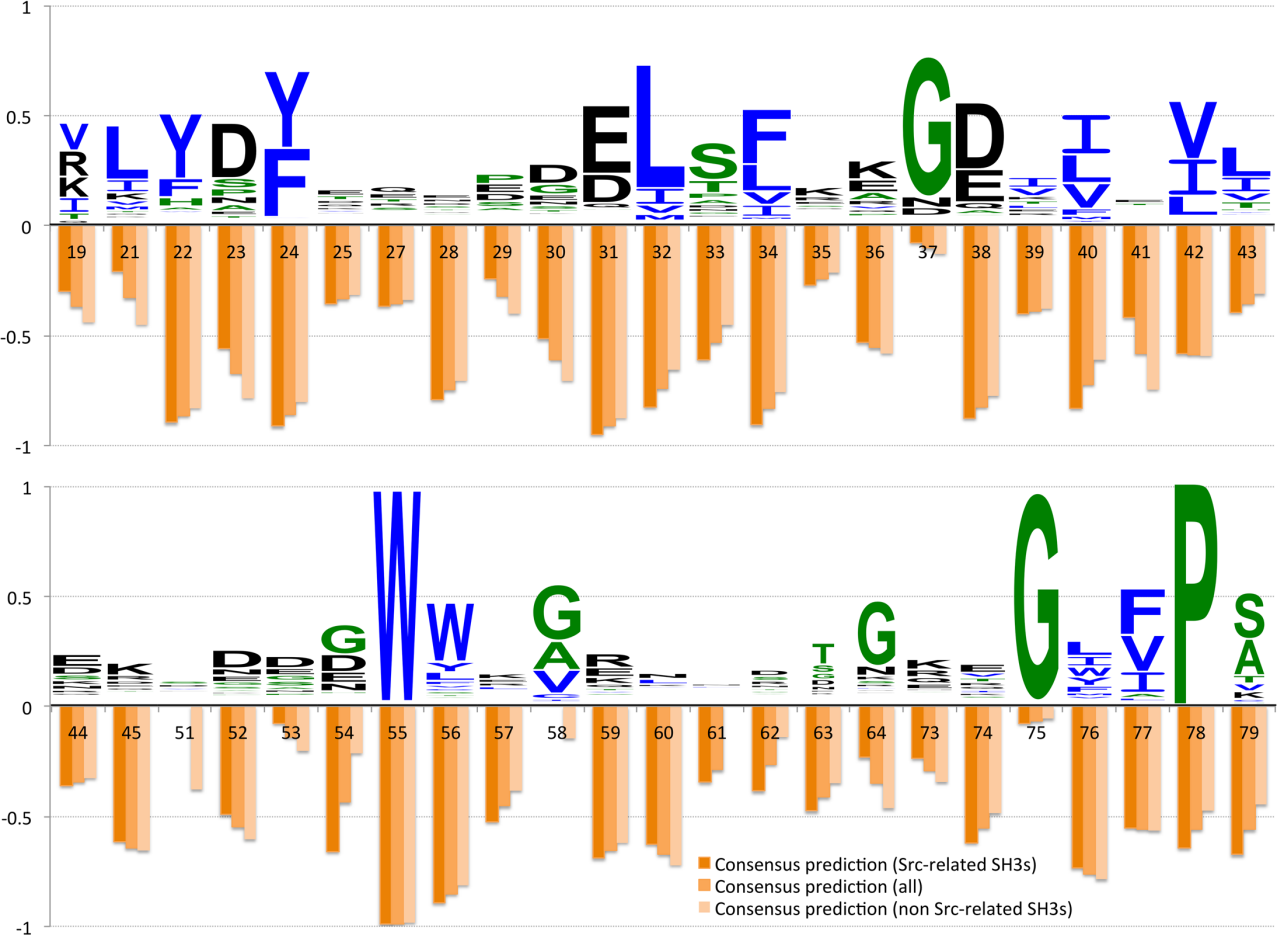
Amino acid conservation in SH3 PFAM family in relation to the consensus rankings. The three consensus rankings are compared to the residue conservation in the SH3 family. The bar chart shows, for 45 positions in the Src SH3 sequence which appear in the PFAM alignment of the complete family (see Material and Methods), the consensus scores (orange bars) derived in this paper and conservation scores derived from the full Pfam alignment of SH3 domains (PF00018) after filtering by 60% of identity (see Material and Methods) (Weblogo). The shading of the orange bars correlates with the distance of the consensus model from Src (i.e. the lighter the bar the further from Src).

**Fig 6:**
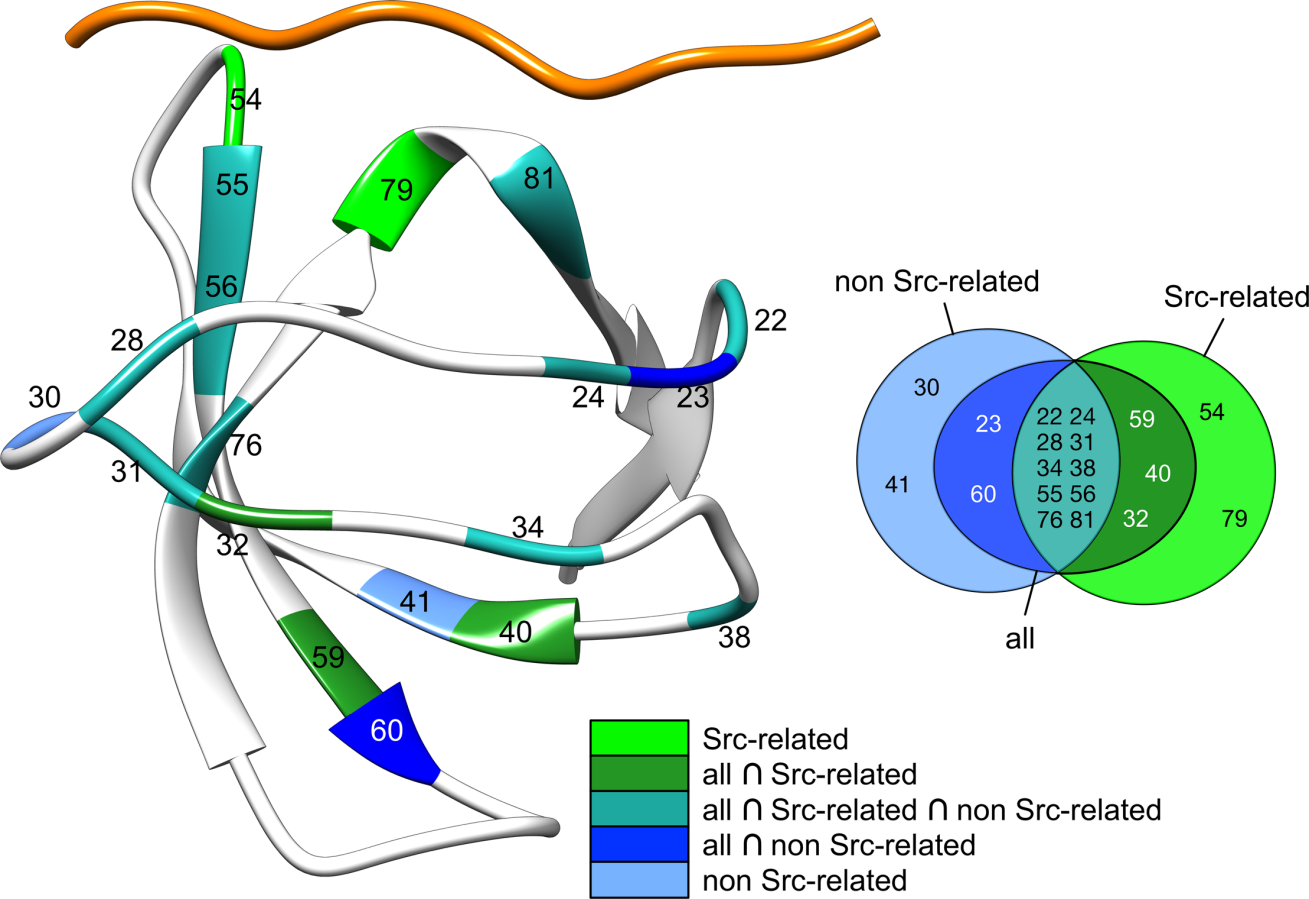
Overlap in consensus rankings. The consensus rankings for the top fifteen residues are mapped on the structure of Src SH3 (same structure used in Fig 1A) with different colors (see legend), e.g. in light cyan are highlighted the residue positions that are consistently ranked in the first 15 positions in all the three consensus rankings, while in light green are highlighted those appearing in the top 15 positions only in the consensus of the SH3 domains from the Src kinases family.

Fig 5 shows the comparison between the conservation profile or Weblogo [51] and the consensus scores (orange bars), normalized between 0 and 1, for the 45 positions in the PFAM alignment for which Src SH3 has no gap (see Material and Methods). The positions are numbered as in the SA of Fig 2. In Fig 6 the 15 top-ranked residues in the three consensus models (see also Table 1) are mapped onto the Src SH3 structure. As such one can visually inspect whether positions highly ranked in the consensus models are also strongly conserved. It should be noted that the MCIT method does not provide insight into the dynamic effects experienced by alanines and glycines [18, 23] due to the fixed orientation of the side-chain in the first and the absence of a side-chain in the second.

Overall some correlation is observed between the consensus results for all residues and the amino acid conservation in SH3 domains (Pearson’s correlation ranging from 0.45 to 0.58, with p-values after Holm-Bonferroni correction [52] always below a confidence level of 0.01).

**Fig 7:**
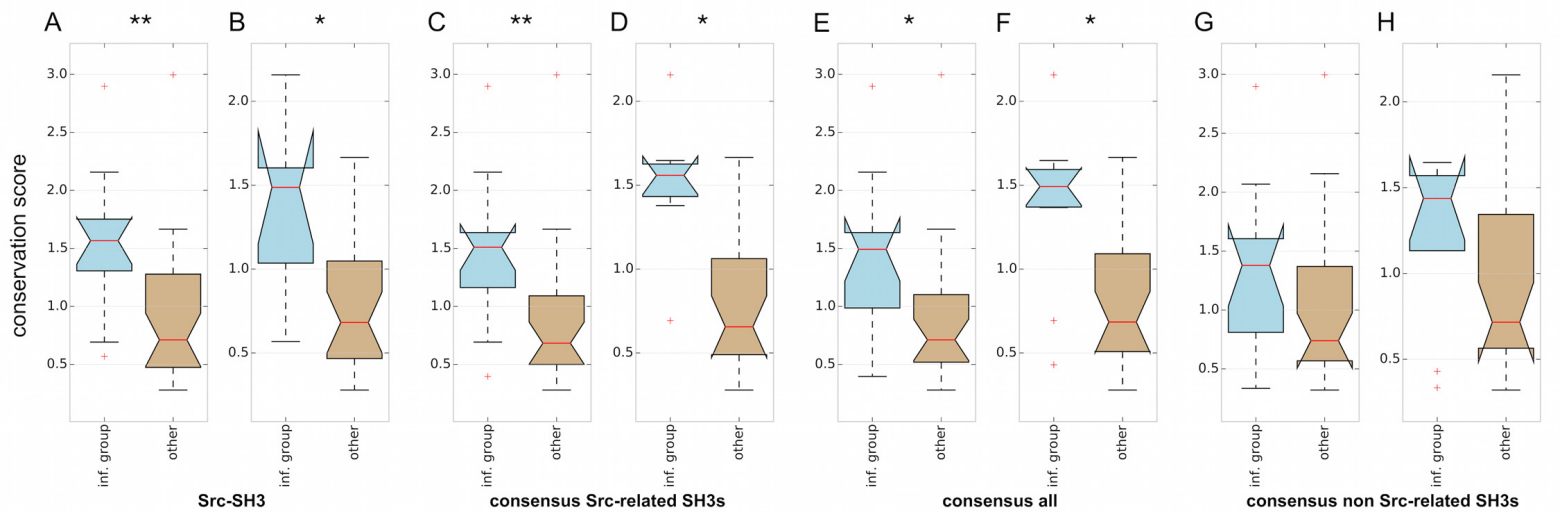
Comparison between predictions and evolutionary conservation. Analysis of the conservation scores of positions predicted to be part of the informative group (inf. group) and all the other positions not ranked in the first 15 positions (other) for Src SH3 (A and B), the consensus of the Src-related SH3 domains (C and D), the consensus of all the eight SH3 domains (E and F), and the consensus of the non Src-related SH3 domains (G and H). The boxplots show the distributions of the conservation scores for the residue positions predicted as part of the informative group and those that are not (A, C, E, and G) and after removal of the binding site residue positions from the distribution (B, D, F, and H). The significance of the difference between the two distributions in each boxplot is evaluated through a Wilcoxon signed-rank test. P-values are corrected applying the Holm-Bonferroni method [52] to account for the multiple comparisons and indicated by ** when below 0.01 and * when below 0.05.

In addition, Fig 7 reveals that the 15 top-ranked residue positions are more conserved than the subsequent ones in the predicted rankings. When removing the binding site residues this conclusion remains unaffected (Fig 7B, 7D, 7F and 7H); the corrected p-values resulting from a Wilcoxon signed-rank test (see Material and Methods) indicate that the distributions are significantly different with 95% confidence level (corrected p-value < 0.05). These results hold for all consensus models (Fig 7A–7F) apart from the one based on the consensus of the non Src-related ones (Fig 7G and 7H), and also for the positions identified solely for the Src SH3 domain (see Fig 7A and 7B). The consensus of non Src-related SH3s is indeed built on variants that have a larger evolutionary distance (see S4 Fig); therefore as expected in that case the separation between the conservation level in the informative group and the conservation level of the other positions is less evident. Nonetheless, the medians of the two boxplots are quite different.

In spite of this general trend, correspondence is not complete. In this way, some less-conserved positions, such as 28, 34, 36, 40, 56 or 76, are predicted to experience strong dynamic changes in the analyzed SH3 variants. These results confirm that conservation does not by itself suffice to establish the importance of a particular residue on the transduction of ligand-induced dynamic changes throughout the protein.

## Discussion

Peptide-induced dynamic changes at the level of the residue side-chains within a protein domain may play a role in the regulation of protein activity. Therefore, the correct prediction of the residues involved in such a transduction process is important. Here we showed first, using the MCIT approach, which residues of Src SH3 are most affected by the binding to a poly-proline class I peptide. This analysis reveals different levels of residues (first-level and second-level subgroups), originating from the binding site, that experience clear changes in their conformational coupling. These changes in conformational dependencies support the knowledge that SH3 domains experience long-range dynamic rearrangements when binding proline-rich ligands. Comparing our predictions to the experimental work of Cordier et al [33, 35] allowed us to show that our results are explainable by an induced fit mechanism[33, 35]: Strain introduced on the H-bonds connecting the β-strands reduces the conformational coupling between sidechains (directly or indirectly). Precision and recall analysis shows that the MCIT approach identifies quite well which residues among all the residues that have H-bonds in Src SH3 experience H-bond effects upon ligand binding. The sidechain coupling hence appears to serve as a proxy for the changes in H-bond lengths at the backbone level. DSC and ITC experiments on the residues in the first- and second-level subgroup show that only mutations in the first-level subgroup affect binding-affinity. These residues, not only are structurally closer to the binding site, but also are mostly located at low-stability regions within the domain, known to be more efficient for signal transduction. In summary, the fact that a residue may experience a dynamic effect, it does not necessarily mean that it can influence the binding affinity.

Second, we analyzed seven additional SH3 domains, i.e. three close homologs found in Lck, Fyn and Hck, and four distant homologs with similarity less than 35% to the Src SH3 domain, with the MCIT approach. The results showed that the similarities in structural predictions are directly correlated to sequence similarity between the SH3 domains: The rankings of dynamic changes for the SH3 domains of Src, Lyn, Fyn and Hck form a cluster, with only a limited correspondence to the distant homologs found in Bin1, Asap1, Eps8l1 and Sptan1. It is worth noticing that it is not completely obvious that one can observe correlations in the predictions as: i) the NMR ensembles of the different SH3 domain variants were produced by different research groups and under different conditions, ii) each ensemble possesses a different degree of structural quality, incorporating for these reasons different levels of noise in the predictions and iii) all SH3 domains interact with different peptides.

Third, three consensus models were constructed that incorporate only the four Src-related SH3 domains, the four distant SH3 domains or all of them at the same time. The consensus models reveal which predicted residues are ranked as most important over the different networks of dynamic changes. In the consensus models including the Src-like SH3 domains, the patterns of residues found in Src SH3 remain rather consistent: most residues in the binding pocket and the residues in the first and second level remain at the top of the list, with a few exceptions. Even more, the induced fit mechanism proposed by Cordier et al [33] is also well supported by the Src-based consensus models. Bigger differences emerge in the consensus model for the non-Src like SH3 domains. These differences occur mostly at the level the second-level subgroup, requiring as a consequence further investigation into the particularities of these distant homologs. Nonetheless, our analysis reveals that the residues predicted to be the most dynamically affected are overall more conserved than less dynamically affected ones. It is important to note that this does not mean that every residue of the most affected is conserved. Some residues like 28, which is located in the n-Src loop, are not very conserved yet are considered to be dynamically important in all consensus models (see Fig 7). These differences may play a crucial role in the specificity of the domain, which requires further investigation.

**Fig 8:**
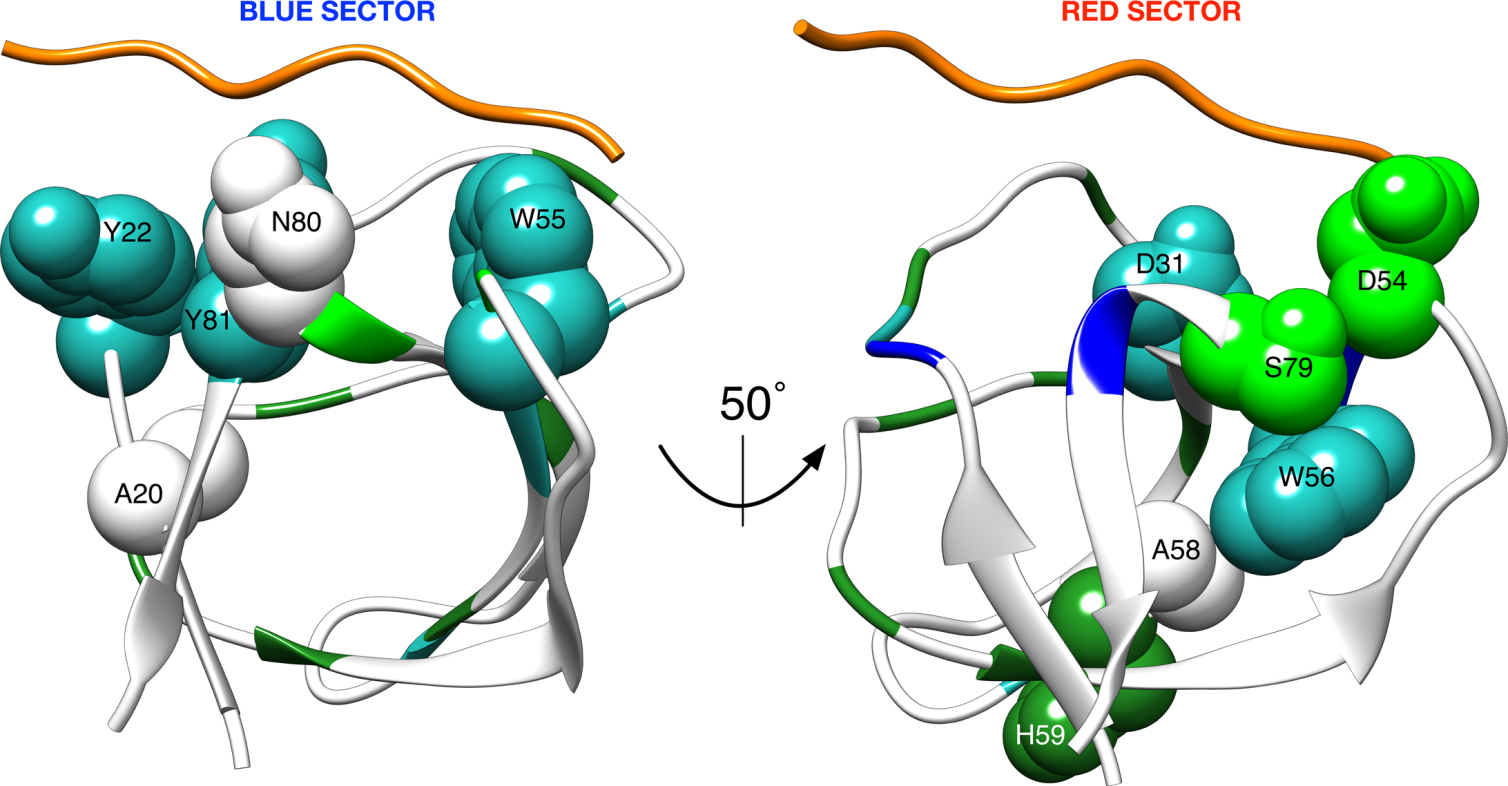
Mapping between the SH3 evolutionary sectors identified in [15] and the consensus predictions. The residues belonging to the two sectors identified in [15] are mapped on the reference structure of Src SH3 (used also in Figs 1A and 7). They are colored in the same way as Fig 6 except for N80, which is not part of the top-ranked residues in the consensus models, and the alanine residues, which cannot be predicted by the MCIT approach. The two orientations of the structure match those reported in figure 7 of [15] to allow for comparison. Residues are labeled following the SA numbering to facilitate comparison with Fig 6.

Interestingly, the consensus prediction for all the SH3 domains reflects well the evolutionary protein sectors that were identified via the Statistical Coupling Analysis (SCA) approach applied on the family of SH3 sequences [15]. In particular, of the two sectors identified in [15], i.e. a red sector composed of (Src SH3 numbering) D31, D54, W56, A58, H59, S79 and a blue sector composed of A20, Y22, W55, N80, Y81, only one residue (N80) is not appearing in our consensus predictions, if we consider that alanine residues cannot be predicted by our approach. Fig 8 shows these results annotated on the Src SH3 structure. Our consensus predictions nonetheless show that other residues might still be relevant having support from at least eight known structures of SH3 domains. These residues, i.e. Y24, T28, F34, E38, Y75 need to be explored further within the context of the evolutionary information and the structural data that is currently available.

Comparing with our consensus models, the SCA results seem to be biased towards the Src-related SH3s. The red sector includes residues D54 and S79 that were ranked among the top fifteen positions only in the consensus of the Src-related SH3s. Indeed, as mentioned before, the relevance of residue D54 in our predictions seems to decrease with the evolutionary distance from Src SH3, as well as the relevance of S79, which also seems to drastically decrease when looking at non Src-related variants. These patterns can be only observed if one looks at the individual predictions and at the consensus predictions at the same time. This capacity of comparing specific and consensus models is exactly the added value provided by our approach. It highlights patterns that may be important for particular subsets of members within the family or even individual ones. Take for instance positions 40 and 41 in the alignment visualized in Fig 2. Whereas position 41 seems to be more relevant in the non-Src related SH3s, the opposite is true for the adjacent position 40 (see also Fig 5). Moreover H59, mutated in R59 in Hck and Eps8l1, is not strongly affected by binding while in Src, despite mutation, this position still shows non-negligible dynamical change upon binding. Is this dissimilarity due to differences in the peptides that bind the structures or may it be explained by the presence (in Hck and Eps8l1) of a lysine in position 57? More of these patterns and hypotheses can easily be formulated when looking at these data, suggesting novel avenues for experimental examination of the functional behavior of these domains.

In the same line we observed significant affinity effects for L32, F34 and W56 in Fig 1D. They are always in the top-ranked set of residues, except in case of the non-Src related consensus model where position 32 falls just outside the top 15 (see Table 1). Nonetheless, only W56 seems to be part of the red SCA sector. It would therefore be interesting to examine the raw SCA data on these residues as to understand why they are not included.

In summary, we showed here through the analysis of different SH3 structures the commonalities and differences at the level of predicted sidechain dynamics induced by peptide binding and showed that the similarities are correlated with an induced fit mechanism previously reported by Cordier et al [33]. Notwithstanding this consistency, the differences between Src-related and non-Src-related SH3 domains requires further exploration as they may provide important insights for understanding how domains differ in regulating protein activity as well as which dynamic patterns are important for the design of domains with specific functionalities[53].

## Materials and Methods

### Structural data used in the analysis

We screened the Protein Data Bank (PDB) for publicly available SH3 NMR ensembles in both ligand-free and ligand-bound forms. We found eight different SH3 domains, which we have used in the computational analysis: a) two Src-SH3 ensembles, corresponding to the ligand-free domain (PDB ID: 1SRM) [54] and the complex with the class I peptide RLP2, RALPPLPRY (PDB ID: 1RLP) [43]; b) two Fyn-SH3 ensembles, i.e. the ligand-free domain (PDB ID: 1NYG) [55] and the complex with the class I peptide PPRPLPVAPG (PDB ID: 1A0N) [56]; c) two Lyn-SH3 ensembles, i.e., the ligand-free domain (PDB ID: 1W1F) [57] and the complex with the peptide WDPGMPTPPLPPRPANLGERQA (PDB ID: 1WA7) [58]; d) the ligand-free domain ensemble (PDB ID: 4HCK) [59] and the ensemble of the complex with peptide HSKYPLPPLPSL (PDB ID: 2OJ2) [60] for Hck-SH3; e) the ligand-free domain ensemble (PDB ID: 2K2M) and the ensemble of the complex with peptide PPVPNPDYEPIR (PDB ID: 2ROL) for Eps8l1-SH3 [61]; f) the ligand-free domain ensemble (PDB ID: 2RQT) and the ensemble of the complex with peptide CIISAMPTKSSRKAKKPAQ (PDB ID: 2RQU) for Asap1-SH3 [62]; g) the ligand-free domain ensemble (PDB ID: 1MUZ) and the ensemble of the complex with peptide LLPTPPLSPSRRSG (PDB ID: 1MV0) for Bin1-SH3 [63]; h) the ligand-free domain ensemble (PDB ID: 2JM9) and the ensemble of the complex with peptide XAPSYSPPPPP (PDB ID: 2JMA) for Sptan1-SH3 [64]. All the proteins studied here belong to human apart from Src and Sptan1, which belong to the chicken.

Prior to sampling, every structure was first energy minimized using the Yasara environment with the Yamber2 force field [65].

### Inferring the changes in dynamics coupling

The predictive approach, originally proposed in [18], has been applied to the SH3 domains as in [23] for the PDZ domains. A Monte-Carlo sampling of the side-chains conformational space is performed on each backbone of the NMR ensembles in the two major states: peptide-free and peptide-bound. During the sampling the FoldX [44] force field is used to calculate the free energy change to determine whether a visited conformational state is energetically favorable. An information-theoretical analysis is then applied on the distributions of the sampled conformations of each pair of residues. This produces two mutual information matrices, one for the peptide-free and one for the peptide-bound ensemble.

Shannon’s mutual information is a measure of the dependency between two random variables [66]; here two residues are considered as two random variables and their realizations are the side-chain conformations they assume during the sampling process.

The change in the dynamic coupling of the residues upon the binding event is then quantified as the absolute difference between the mutual information coupling of the side-chain conformation in the two states. Details related to this approach can be found in [23], together with the description of a number of additional pre-processing and filtering steps that we introduced to improve the mutual information calculations.

The *informative group* is derived from the matrix of dynamical changes by applying the Cluster Affinity Search Technique (CAST) [67], which aims at identifying the cliques of most connected vertices in a graph. Here our aim is to identify the groups of residues that are most affected by the binding event.

### Protein and peptide samples

The plasmid pET15b containing the chicken c-Src-SH3 domain gene was a generous gift from Dr. E. Freire (Johns Hopkins University). The genes encoding for the c-Src-SH3 mutants were obtained by site directed mutagenesis using the QuickChange Site-Directed-Mutagenesis Kit (Stratagene) taking the WT c-Src-SH3 plasmid as template or purchased from Top Gene Technologies, Canada. WT and mutants of c-Src-SH3 domain were expressed and purified as previously described for the wild type protein [68]. The peptide RLP2 (Ac-RALPPLPRY-NH2) was bought from Peptide 2.0 (Piscataway, USA) and JPT (Berlin, Germany) with a purity > 95%. Protein concentration was determined by absorbance at 280 nm using an extinction coefficient of 16500 M^-1^·cm^-1^. Peptide concentration was determined by absorbance at 274 nm using an extinction coefficient of 1400 M^-1^·cm^-1^.

### Differential scanning calorimetry

Differential scanning calorimetry was performed on a VP-DSC microcalorimeter (MicroCal, USA) at a heating rate of 1.5 K·min^1^ using protein concentrations within the range of 0.3–0.5 mg·mL^-1^. The temperature dependence of the molar partial heat capacity (Cp) of the SH3 domains was calculated from the DSC data and analyzed using Origin 6.1 (OriginLab). Cp curves were fitted by a non-linear least-squares method using the two-state unfolding model as described elsewhere [69].

### Isothermal titration calorimetry

Isothermal titration calorimetry was performed using a high-precision VP titration calorimetric system (Microcal Inc., Northampton, MA) as previously described [37]. The c-Src-SH3 domain variants in the calorimetric cells (between 50 μM and 122 μM) were titrated with the ligand at about 2 mM. Titrations were made using a profile of injection volumes from 3 μL to 20 μL to define the titration curve more clearly. The heat produced by the binding reaction between the Src-SH3 domain and the peptide ligand was calculated as the difference between the heat of reaction and the corresponding heat of dilution, as obtained from independent titrations of the peptide ligand into the buffer. The resulting binding isotherms were analyzed by non-linear least-square fittings of the experimental data to a model corresponding to a single set of identical sites, as described before [37].

### Structural alignment and phylogenetic tree

By using the T-Coffee structural alignment program [70], we produced the alignment of the SH3 domains in Fig 2. We further checked the correctness of this alignment by manual inspection of the structural alignment produced by using the MUSTANG algorithm [71] as implemented in YASARA (http://www.yasara.org). By doing so we identified regions where the structural alignment can be considered more reliable, which are highlighted in orange in Fig 2.

### Alignment score and correlation of the predictions

The pairwise alignment scores of each SH3 domain with Src SH3 are computed starting from the structural alignment in Fig 2 by using a BLOSUM 62 score matrix and a gap extension and a gap open penalty of, respectively, -1 and -5 (x-axis in Fig 3A).

By repeating the clustering by CAST several times with decreasing threshold, it is possible to observe which residues are included in the relevant clusters and, in doing so, to obtain a ranking of the residues in the domain based on the magnitude of the dynamical change induced by peptide binding. In other words, each residue is associated to an affinity threshold value at which it becomes part of a clique. These values associated to each residue (that we call predictions) determine a ranking. The y-axis in Fig 3A reports the Spearman’s rank correlation computed between the predictions of each pair of SH3 domain. In practice this correlation is computed in the following way: we first transform the predictions for each SH3 domain into residue ranks (the standard procedure used in computing the Spearman’s rank correlation is used here: values are assigned a position, i.e. a rank, in ascending order and the rank of identical values corresponds to an average of their positions in this ordering); then for the subset of residues that correspond in the structural alignment and are not aligned to an alanine residue, we compute the Pearson’s correlation between the ranked predictions, since the Spearman’s rank correlation corresponds by definition to the Pearson’s correlation of the ranked variables.

### Consensus prediction

We computed a consensus ranking of the residue predictions for the eight different SH3 domains and for the four SH3 domains belonging to the Src family of kinases. The consensus rank of a residue is computed as a weighted sum of the ranks of the residue in the different SH3 domain related predictions:

$${consensus\_rank}_{i}=\frac{\sum_{j=1}^{n}\frac{1}{max({ranking}_{j})}∙{rank}_{ij}}{n}$$ (1)

where *i* is a residue position in the multiple alignment of Fig 2 and *j* the index of one SH3 domain, with *n* being the number of SH3 domains used to build the consensus ranking, either 4 or 8 in this paper. The maximum of a ranking corresponds to the length of the SH3 domain when there are no rank ties at one extreme of the ranking; otherwise it corresponds to the average of the positions of the identical values. Table 1 reports a summary of the three consensus rankings. Note that positions that in the consensus ranking contain more than one fourth of missing information due to the presence of alanine residues or gaps in the structural alignment of Fig 2 are not reported in the table.

### Analysis of conservation and relation to the predictions

To perform this analysis we started from the full Pfam alignment of SH3 domains (PF00018) containing 10749 sequences. We filtered the alignment by 60% identity, obtaining an alignment of 1242 SH3 domain sequences. From this filtered alignment we computed the conservation scores as in the Weblogo software [51]. The Pfam alignment resulted in 48 positions corresponding to Src SH3. In Fig 5 we do not report predictions for the positions containing more than one fourth of missing information due to the presence of alanine residues or gaps in the structural alignment of Fig 2, as we previously did for Table 1 and Fig 5. This also applies to all the subsequent analyses of correlation between predictions and conservation. In addition to evaluate the correlation between predictions and conservation we also removed positions having glycine residues in at least half of the sequences, since the relevance of glycine residues in side-chain dynamics is obvious but the conservation patterns vary a lot so that they could affect the correlation. In particular, as it can be observed from Fig 5 the SH3 domain family has at least two very well conserved glycine positions (37, 75).

The boxplots in Fig 7 were produced by considering the conservation score distribution over the group of top fifteen residues (A-H, left) and over the group of subsequent ones in the predicted ranking (A-H, right) for Src SH3 (A and B), the consensus of the Src-related SH3 domains (C and D), the consensus of all the eight SH3 domains (E and F), and the consensus of the non Src-related SH3 domains (G and H). In B, D, F, and H we performed the same analysis as in A, C, E, and G, respectively, after removal of the binding site residues. We identified as binding site residues all the residues that in all the considered SH3 variants satisfy the following criterion: they are at distance less than 4 Angstrom from the residues of the peptide (as determined by YASARA) in at least 60% of the structures in each NMR ensemble. For instance in the case of the consensus of all the eight SH3s, the resulting list is composed of positions 24, 28, 55, 76, 78 in Fig 6. The significance of the difference between the two distributions in each boxplot is evaluated through a Wilcoxon signed-rank test. P-values are corrected applying the Holm-Bonferroni method [52] to account for the multiple comparisons.

## Supporting Information

S1 Text Additional in vitro results. (DOCX)

S1 Fig Titration thermograms for c-Src WT and the mutations of residues F18, S26, F34, L40 and N45. (TIF)

S2 Fig Titration thermograms for the mutations of residues W56, H59, R73 and I77. (TIF)

S3 Fig L32V, F34I and W56L mutations and their effect on the phosphorylation of Src. Effect of the mutants W56L, F34I, and L32V (boxplot in gray) on the fraction of phosphorylated protein and the total amount of protein relative to the same ratio in the wild type (see S5 Table). The positions of the mutants within the Src sequence (Uniprot ID: P00523) are shown within brackets on the x-axis. Each mutant was tested 4 times, corresponding to the four red circles per mutant in the figure. The mutants L32V shows an effect similar to mutating the binding pocket residue D31, although both were overexpressed. F34I and W56L reduce the relative phosphorylation levels slightly with an expression level similar to that of the wild type.
(TIF)

S4 Fig Phylogenetic tree of the SH3 domains analyses in this study. The Src-related SH3 domains are encircled in red. The phylogenetic tree was produced starting from the SA in Fig 3, by using ClustalW2-Phylogeny (http://www.ebi.ac.uk/Tools/phylogeny/clustalw2_phylogeny/) with default parameter setting (neighbor-joining algorithm).
(TIF)

S1 Table Annotation of residues experiencing H-bond changes on the twenty residues ranked at the top in Table 1. This table provides for the Src SH3 predictions as well as the three consensus models an annotated table of the twenty residues ranked as most important by the MCIT approach. For each of the four models, we provide the name and numbering of the residue in the Cordier et al article [33] and the (consensus) residue plus the location of the residue following the numbering of Fig 2. Residues annotated with a ‘*’ are directly involved in an H-bond in the Cordier et al article and those annotated with ‘†’ are directly next to one in the Src SH3 sequence. The residues in brackets are missing data in [33] due to spectral overlaps and the remaining residues do not fit any of the previous three classes. As can be seen, the majority of residues are either annotated, revealing that our predictions are almost always linked to residues experiencing the lengthening or shrinking of their H-bonds.
(DOCX)

S2 Table Precision and recall analysis of the predicted rankings relative the H-bonds effects. This table includes all residues (first column) that are part of an H-bond as discussed in [33]. Alanines and glycines involved in H-bonds were excluded, as the MCIT approach cannot make any predictions about those amino acids. Each residue (column 2) is annotated with the change it experiences upon binding the peptide: +/- indicating a lengthening/shrinking of the H-bond (which are the true positives, TP) and ‘0‘ indicating no effect (which are the true negatives, TN). For each of these residues the MCIT predicted rank for the Src SH3 structure is added and the table is sorted on this rank. Using this data and a given rank threshold, one can determine precision (TP/(TP+FP)) and recall (TP/(TP+FN)). From a threshold of 15 on, recall starts to include more than 50% of the residues that experience an effect in the H-bond wherein they are involved. Yet from that point the precision, i.e. the number of correctly identified H-bond residues, starts to decrease. Nonetheless, even for a threshold up to 25 do we obtain a precision of more than 90%.
(DOCX)

S3 Table Analysis of Src SH3 binding affinities through ITC. Thermodynamic binding affinity to peptide RLP2. A * indicates experiment made by fluorescence titration only.
(DOC)

S4 Table Analysis of Src SH3 stability upon mutation. Folding parameters of c-Src SH3 domains and diverse mutants, obtained from a multiple curve fitting of all DSC experiments, considering common C_pN_ and C_pU_ functions for Src wt and all mutants analysed.
(DOC)

S5 Table Data for in cell experiments. Experiments were repeated four times. Both the raw results, the transformations relative to the WT and the averages (and standard deviations) are reported.
(DOCX)
