# Supplementary material for: From Binding-Induced Dynamic Effects in SH3 Structures to Evolutionary Conserved Sectors
Source: PLoS Comput Biol. 2016 May 23;12(5):e1004938. doi: 10.1371/journal.pcbi.1004938 (PMC4877006; doi:10.1371/journal.pcbi.1004938)
Supplement: S1 Text — (DOCX) [file pcbi.1004938.s001.docx]

***Supporting Information for***

**From binding-induced dynamic effects in SH3 structures to evolutionary conserved sectors**

Ana Zafra Ruano^1^^¶^, Elisa Cilia^2,3¶^, José R. Couceiro^4,5¶^, Javier Ruiz Sanz^1^, Joost Schymkowitz^4,5^, Frederic Rousseau^4,5^, Irene Luque^1^ and Tom Lenaerts^2,3,6,*^

## Additional experimental in vitro results

We examined also how the mutants L32V, F34I and W56L, which had the strongest affinity effects, translate into Src activity. We measured activity here determining whether these mutants induce more phosphorylation activity than the wild type (see Supporting Methods). In the absence of a stimulus, Src adopts an auto-inhibited conformation, stabilized by intra-molecular interactions of the SH2 and SH3 domains that lock the kinase domain in an inactive state [1, 2]. The state of activation of the Src protein can be monitored by the phosphorylation of tyrosine 416 (Y416, following the numbering in Uniprot ID: P00523), a residue available to kinases only when the protein is in the open conformation.

It has been described that mutation of the binding pocket of the SH3 domain (D31N) shows an effect on the intra-molecular regulation of the activity of Src. As can be seen in S3 Fig, the mutant D31N results in an increase in the phosphorylation of Y416, although certainly not as pronounced as when caused by the Y527F mutation, where Y527 is the tyrosine located in the tail of Src [3]. Together with the results for the WT, these three cases serve as controls to examine the impact of the L32V, F34I and W56L mutants (see S3 Fig and also the raw data in S3 Table).

Although the L32V mutant is destabilizing and has a strong effect on binding the RALPPLPRY peptide, the effects on the phosphorylation activity are not as pronounced: there is a slight increase in the phosphorylation of Y416, at a level equivalent to the change induced by mutating the binding-pocket residue D31. Upon examining the raw data, we observed that in both cases the mutants are over-expressed, which might explain the up-regulation in comparison to the wild type. Nonetheless, the relevance of D31 was reported before [3]. The two remaining mutants W56L and F34I on the other hand seem to reduce the activity of Src phosphorylation slightly, without being overexpressed (Note that significance tests cannot be performed here due to the limited number of samples). Considering that the changes in the phosphorylation state of Y416 arise from the disruption of both of intra- and inter-molecular contacts, the results for W56L and F34I indicate that these residues may have a role to play in the activity of Src. To confirm this hypothesis, additional repeats of the same experiment as well as more extensive screening of the other residues shown in Fig 1C are required. Moreover, a new stability/affinity study should be performed in light of the peptide representing the SH2/Kinase linker.

## Supplementary Methods

##

**Phosphorylation experiments - Antibodies and plasmids**

Anti-Src (32G6) monoclonal and Phospho-Src Family (Tyr416) polyclonal rabbit antibodies were purchased from Cell Signaling Technology. Retroviral vectors expressing wt chicken c-Src or Y416F and Y527F mutants were purchased from Addgene (plasmids 13665, 13662 and 13665 respectively). Point mutations in wt Src (D99N, L100V, F102I and W119L) were introduced by oligonucleotide primer–based PCR mutagenesis using Pwo DNA polymerase (Roche). Sequence analysis indicated that no additional mutations were introduced.

**Phosphorylation experiments: Cell lysis, immunoprecipitation and western-blot**.

SYF cells were cultured in Dulbecco’s modified Eagle’s medium (DMEM) supplemented with 10% fetal bovine serum (FBS) and 1% penicillin/streptomycin at 37º C in an atmosphere of 5% CO_2_. All cell culture reagents were obtained from Invitrogen. Transfections were carried out in 10 cm dishes with Fugene (Promega) following the manufacturer’s recommendations. 48 h after transfection, cells were washed with cold PBS, and lysed in a lysis buffer containing NP-40 (50 mM Tris [pH 7.5], 150 mM NaCl, 2 mM EDTA, 1% NP-40, 0.5% sodium deoxycholate, Complete protease inhibitor tablets (Roche) and Halt Phosphatase Inhibitor cocktail (Thermo Scientific)). Cell debris was cleared by centrifuging at 12,000 xg at 4°C for 10 min, and supernatants were taken as the cell lysate. Immunoprecipitations were performed by first incubating 1,5 μg of Anti-Src antibody with the cell lysates for 3 h at RT. Protein G-immobilized Sepharose beads (Pierce) were then added and incubated for 2 h at 4°C. Immunoprecipitates were washed four times with the appropriate lysis buffer and resuspended in electrophoresis sample buffer. SDS-polyacrylamide gel electrophoresis (PAGE) and Western blotting were performed according to standard procedures. Following incubation with Anti-Src or Anti-phospho-Src primary antibodies, goat anti-rabbit secondary antibodies conjugated to horseradish peroxidise (Promega) were used and immunoreactive proteins were visualized with ECL chemiluminescence kit (Pierce). Positive bands were quantified with Quantity One Sofware (Bio-Rad).

## References

1. Sicheri F, Kuriyan J. Structures of Src-family tyrosine kinases. Current opinion in structural biology. 1997;7(6):777-85. Epub 1998/01/22. PubMed PMID: 9434895.

2. Kaplan KB, Bibbins KB, Swedlow JR, Arnaud M, Morgan DO, Varmus HE. Association of the amino-terminal half of c-Src with focal adhesions alters their properties and is regulated by phosphorylation of tyrosine 527. The EMBO journal. 1994;13(20):4745-56. Epub 1994/10/17. PubMed PMID: 7525268; PubMed Central PMCID: PMC395413.

3. Cary LA, Klinghoffer RA, Sachsenmaier C, Cooper JA. SRC catalytic but not scaffolding function is needed for integrin-regulated tyrosine phosphorylation, cell migration, and cell spreading. Mol Cell Biol. 2002;22(8):2427-40. Epub 2002/03/23. PubMed PMID: 11909938.
