# Supplementary figures and images for: From Binding-Induced Dynamic Effects in SH3 Structures to Evolutionary Conserved Sectors
Source: PLoS Comput Biol. 2016 May 23;12(5):e1004938. doi: 10.1371/journal.pcbi.1004938 (PMC4877006; doi:10.1371/journal.pcbi.1004938)

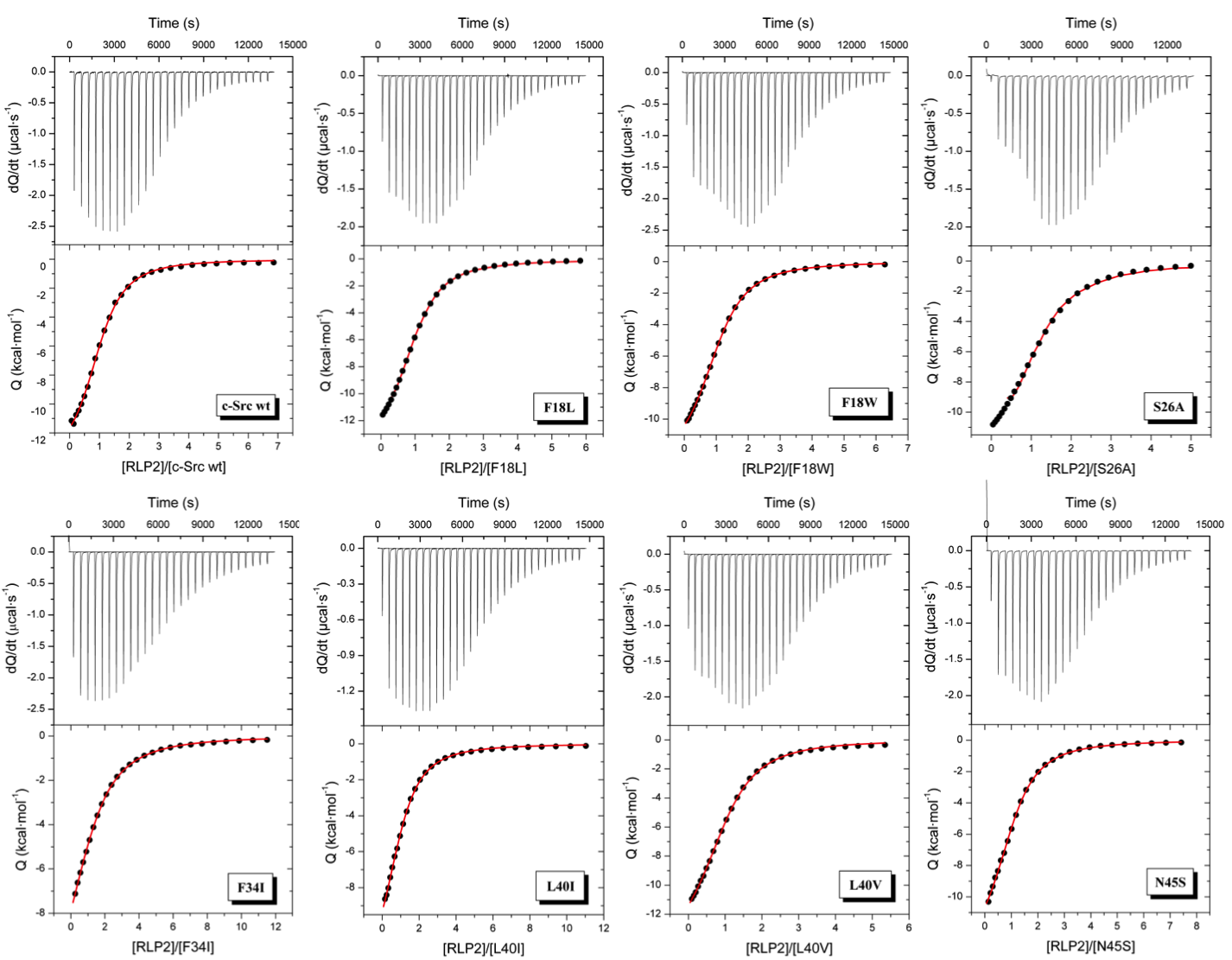

Supplement: S1 Fig — (TIF) [file pcbi.1004938.s002.tif]

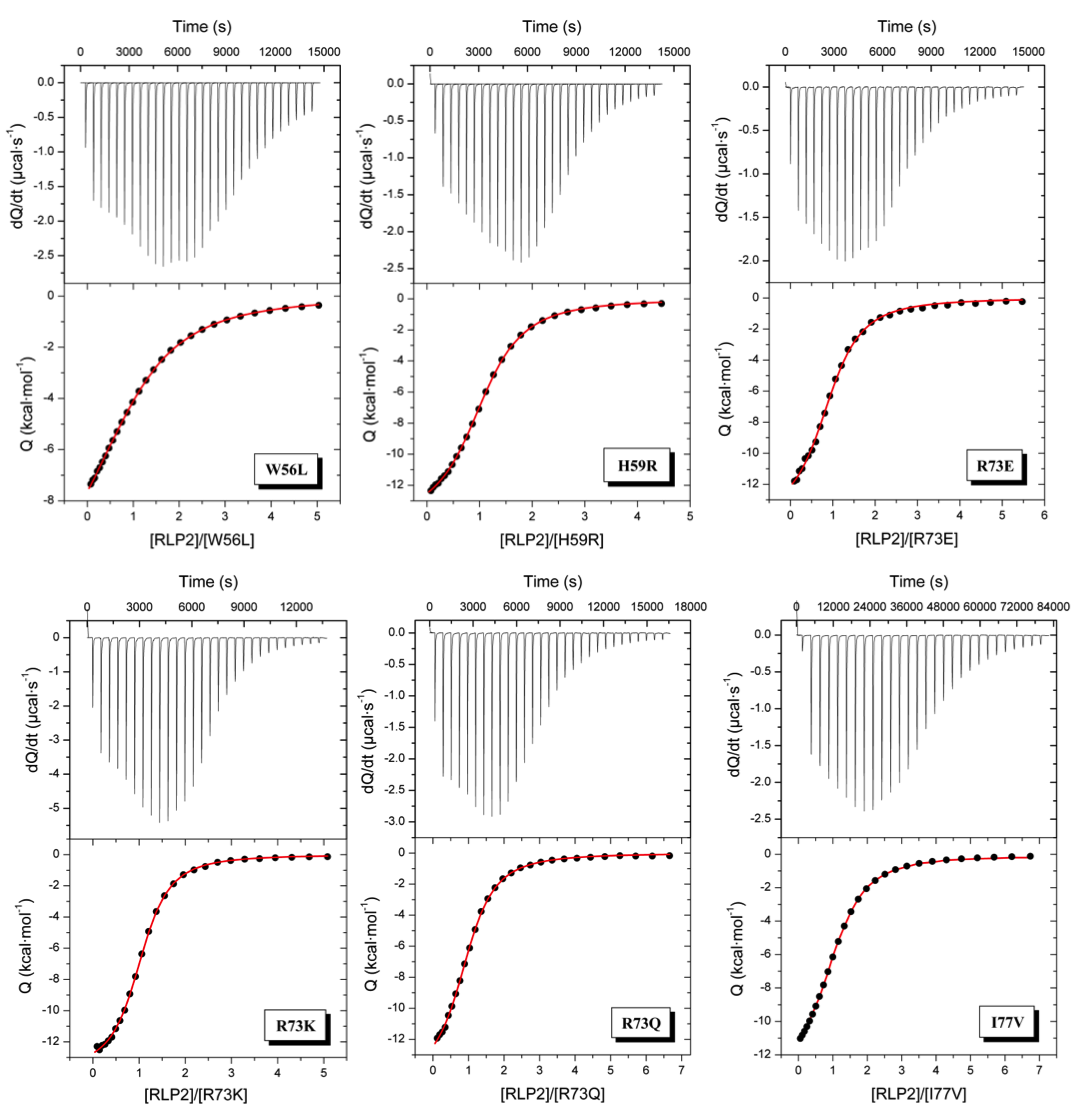

Supplement: S2 Fig — (TIF) [file pcbi.1004938.s003.tif]

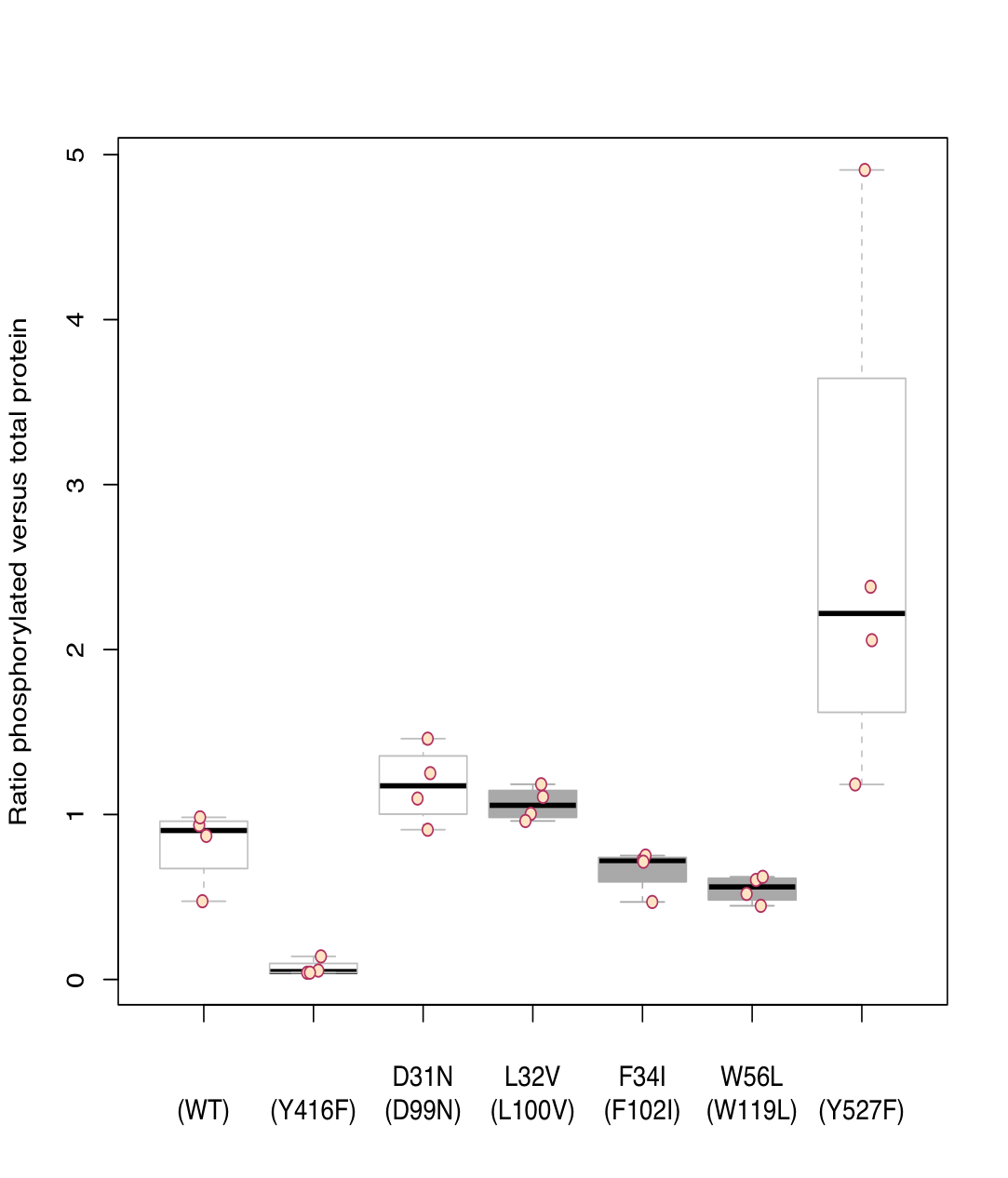

Supplement: S3 Fig — Effect of the mutants W56L, F34I, and L32V (boxplot in gray) on the fraction of phosphorylated protein and the total amount of protein relative to the same ratio in the wild type (see S5 Table). The positions of the mutants within the Src sequence (Uniprot ID: P00523) are shown within brackets on the x-axis. Each mutant was tested 4 times, corresponding to the four red circles per mutant in the figure. The mutants L32V shows an effect similar to mutating the binding pocket residue D31, although both were overexpressed. F34I and W56L reduce the relative phosphorylation levels slightly with an expression level similar to that of the wild type. (TIF) [file pcbi.1004938.s004.tif]

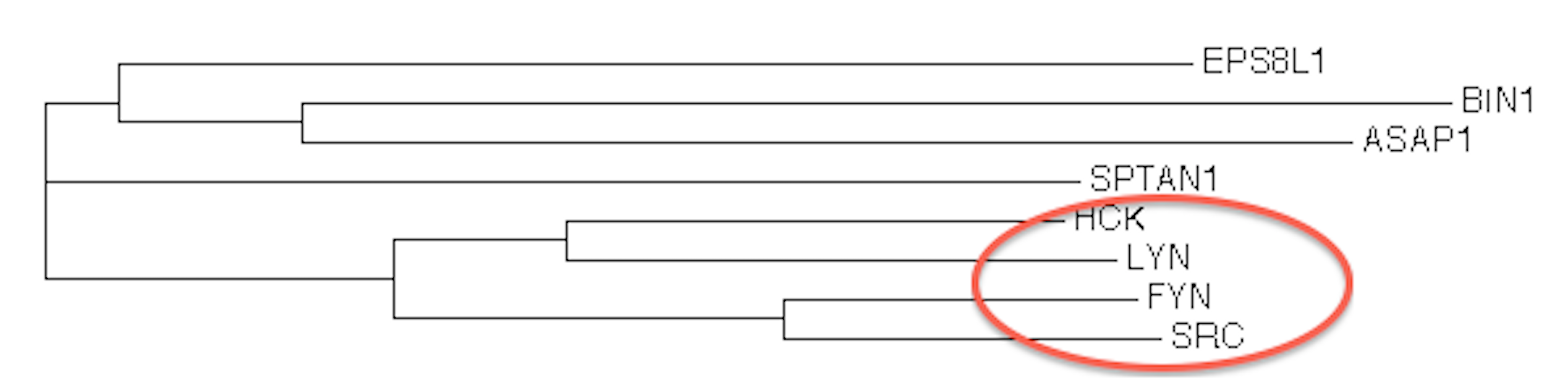

Supplement: S4 Fig — The Src-related SH3 domains are encircled in red. The phylogenetic tree was produced starting from the SA in Fig 3, by using ClustalW2-Phylogeny (http://www.ebi.ac.uk/Tools/phylogeny/clustalw2_phylogeny/) with default parameter setting (neighbor-joining algorithm). (TIF) [file pcbi.1004938.s005.tif]
