# Supplementary material for: From Binding-Induced Dynamic Effects in SH3 Structures to Evolutionary Conserved Sectors
Source: PLoS Comput Biol. 2016 May 23;12(5):e1004938. doi: 10.1371/journal.pcbi.1004938 (PMC4877006; doi:10.1371/journal.pcbi.1004938)
Supplement: S1 Table — This table provides for the Src SH3 predictions as well as the three consensus models an annotated table of the twenty residues ranked as most important by the MCIT approach. For each of the four models, we provide the name and numbering of the residue in the Cordier et al article [33] and the (consensus) residue plus the location of the residue following the numbering of Fig 2. Residues annotated with a ‘*’ are directly involved in an H-bond in the Cordier et al article and those annotated with ‘†’ are directly next to one in the Src SH3 sequence. The residues in brackets are missing data in [33] due to spectral overlaps and the remaining residues do not fit any of the previous three classes. As can be seen, the majority of residues are either annotated, revealing that our predictions are almost always linked to residues experiencing the lengthening or shrinking of their H-bonds. (DOCX) [file pcbi.1004938.s006.docx]

|  | **Residues involved in H-bond in Src SH3** | **Src Sh3** | | **Residues involved in H-bond in Src SH3** | **Consensus Src-related** | | **Residues involved in H-bond in Src SH3** | **Consensus all** | | **Residues involved in H-bond in Src SH3** | **Consensus non-Src related** | |
| --- | --- | --- | --- | --- | --- | --- | --- | --- | --- | --- | --- | --- |
| **Ranking** | **Cordier et al numbering** | **AA** | **#** | **Cordier et al numbering** | **AA** | **#** | **Cordier et al numbering** | **AA** | **#** | **Cordier et al numbering** | **AA** | **#** |
| 1 | W39 (c.t. D38-S55)† | W | 55 | W39† | W | 55 | W39† | W | 55 | W39† | W | 55 |
| 2 | Y57* | Y | 81 | Y57* | y | 81 | Y57* | y | 81 | D20† | e | 31 |
| 3 | (F23) | F | 34 | D20† | e | 31 | D20† | e | 31 | Y57* | y | 81 |
| 4 | H43* | H | 59 | (Y13) | y | 24 | Y11† | Y | 22 | Y11† | Y | 22 |
| 5 | F7* | F | 18 | (F23) | f | 34 | (Y13) | y | 24 | W40* | W | 56 |
| 6 | (Y13) | Y | 24 | Y11† | Y | 22 | W40* | W | 56 | (Y13) | y | 24 |
| 7 | D20 (c.t. L21-Y52)† | D | 31 | W40* | W | 56 | (F23) | f | 34 | Y52* | y | 76 |
| 8 | E27 (c.t. L29-F7) | E | 38 | E27 | e | 38 | E27 | e | 38 | D12 | D | 23 |
| 9 | L29* | L | 40 | L29* | l | 40 | Y52* | y | 76 | E27 | e | 38 |
| 10 | W40* | W | 56 | L21* | L | 32 | T17 | h | 28 | (F23) | f | 34 |
| 11 | L21* | L | 32 | T17 | h | 28 | L21* | L | 32 | Q30* | q | 41 |
| 12 | T6* | T | 17 | Y52* | y | 76 | L29* | l | 40 | S61* | l | 85 |
| 13 | I53* | I | 77 | H43* | r | 59 | S44* | s | 60 | S44* | s | 60 |
| 14 | N34 (c.t. N33-L41)† | N | 45 | S55* | s | 79 | D12 | D | 23 | T19 | d | 30 |
| 15 | D38* | D | 54 | D38* | e | 54 | H43* | r | 59 | T17 | h | 28 |
| 16 | S55* | S | 79 | P54† | P | 78 | N34† | e | 45 | N34† | e | 45 |
| 17 | P54 (c.t. S55-D38)† | P | 78 | S44* | s | 60 | T6* | i | 17 | L21* | L | 32 |
| 18 | S15 | S | 26 | T50† | e | 74 | T19 (c.t. L21-Y52) | d | 30 | T6* | i | 17 |
| 19 | D12 (c.t. Y13-F23) | D | 23 | N34† | e | 45 | I31* | v | 42 | H43* | r | 59 |
| 20 | Y11 (c.t. L10-Y57)† | Y | 22 | S22 (c.t. L21-Y52)† | s | 33 | Q30* | q | 41 | L29* | l | 40 |
|  |  |  |  |  |  |  |  |  |  |  |  |  |
|  |  |  |  |  |  |  |  |  |  |  |  |  |
