# Supplementary material for: From Binding-Induced Dynamic Effects in SH3 Structures to Evolutionary Conserved Sectors
Source: PLoS Comput Biol. 2016 May 23;12(5):e1004938. doi: 10.1371/journal.pcbi.1004938 (PMC4877006; doi:10.1371/journal.pcbi.1004938)
Supplement: S2 Table — This table includes all residues (first column) that are part of an H-bond as discussed in [33]. Alanines and glycines involved in H-bonds were excluded, as the MCIT approach cannot make any predictions about those amino acids. Each residue (column 2) is annotated with the change it experiences upon binding the peptide: +/- indicating a lengthening/shrinking of the H-bond (which are the true positives, TP) and ‘0‘ indicating no effect (which are the true negatives, TN). For each of these residues the MCIT predicted rank for the Src SH3 structure is added and the table is sorted on this rank. Using this data and a given rank threshold, one can determine precision (TP/(TP+FP)) and recall (TP/(TP+FN)). From a threshold of 15 on, recall starts to include more than 50% of the residues that experience an effect in the H-bond wherein they are involved. Yet from that point the precision, i.e. the number of correctly identified H-bond residues, starts to decrease. Nonetheless, even for a threshold up to 25 do we obtain a precision of more than 90%. (DOCX) [file pcbi.1004938.s007.docx]

| All H-bond residues | Changed H-bond residues | Src numbering in paper | Src ranking (see Table 1) | Threshold | Precision | Recall |
| --- | --- | --- | --- | --- | --- | --- |
| Y57 | Y57+ | Y81 | 2 |  |  |  |
| F23 | missing | F34 | 3 |  |  |  |
| H43 | H43+ | H59 | 4 |  |  |  |
| F7 | F7+ | F18 | 5 |  |  |  |
| Y13 | missing | Y24 | 6 |  |  |  |
| L29 | L29+ | L40 | 9 |  |  |  |
| W40 | W40+ | W56 | 10 | **10** | 1,00 | 0,33 |
| L21 | L21- | L32 | 11 |  |  |  |
| T6 | T6+ | T17 | 12 |  |  |  |
| I53 | I53+ | I77 | 13 |  |  |  |
| D38 | D38+ | D54 | 15 | **15** | 1,00 | 0,52 |
| S55 | S55+ | S79 | 16 |  |  |  |
| D12 | 0 | D23 | 19 | **20** | 0,92 | 0,57 |
| S44 | S44+ | S60 | 23 | **25** | 0,93 | 0,62 |
| T35 | 0 | T51 | 26 |  |  |  |
| Y52 | Y52- | Y76 | 30 | **30** | 0,88 | 0,67 |
| Q30 | Q30+ | Q41 | 31 |  |  |  |
| L41 | L41- | L57 | 32 |  |  |  |
| R28 | 0 | R39 | 33 |  |  |  |
| I31 | I31- | I42 | 34 |  |  |  |
| V8 | 0 | V19 | 35 |  |  |  |
| L45 | 0 | L61 | 36 |  |  |  |
| K25 | 0 | K36 | 37 |  |  |  |
| T19 | 0 | T30 | 39 |  |  |  |
| V32 | 0 | V43 | 40 | **40** | 0,68 | 0,81 |
| S61 | S61+ | S87 | 42 |  |  |  |
| N33 | N33- | N44 | 43 |  |  |  |
| Q49 | Q49+ | R73 | 44 |  |  |  |
| L10 | L10+ | L21 | 46 |  |  |  |
