# Supplementary material for: From Binding-Induced Dynamic Effects in SH3 Structures to Evolutionary Conserved Sectors
Source: PLoS Comput Biol. 2016 May 23;12(5):e1004938. doi: 10.1371/journal.pcbi.1004938 (PMC4877006; doi:10.1371/journal.pcbi.1004938)
Supplement: S3 Table — Thermodynamic binding affinity to peptide RLP2. A * indicates experiment made by fluorescence titration only. (DOC) [file pcbi.1004938.s008.doc]

| **Protein** | **K_d_ (25 ºC)**  **(µM)** | **∆G_b_ (25 ºC)**  **(kcal/mol)** | **∆∆G_b_ (25 ºC)**  **(kcal/mol)** | **∆H_b_ (25 ºC)**  **(kcal/mol)** | **∆∆H_b_ (25 ºC)**  **(kcal/mol)** |
| --- | --- | --- | --- | --- | --- |
| c-Src wt | 13.8±0.6 | -6.63 ±0.03 | 0 | -14.2±0.2 | 0 |
| c-Src F18L | 13.5±0.3 | -6.64±0.01 | -0.01±0.03 | -14.5±0.1 | -0.3±0.2 |
| c-Src F18W | 20.2±0.3 | -6.40±0.01 | 0.23±0.03 | -13.0±0.1 | 1.2±0.2 |
| c-Src S26A | 18.8±1.4 | -6.45±0.05 | 0.18±0.06 | -13.8±0.5 | 0.4±0.5 |
| c-Src L32V^*^ | 269±29 | -4.87±0.06 | 1.76±0.07 | - | - |
| c-Src F34I | 52.6±1.2 | -5.84±0.01 | 0.79±0.03 | -13.6±0.3 | 0.6±0.3 |
| c-Src L40I | 20.6±0.6 | -6.39±0.02 | 0.24±0.04 | -13.8±0.3 | 0.4±0.4 |
| c-Src L40V | 20.5±0.6 | -6.39±0.02 | 0.24±0.04 | -14.8±0.2 | -0.6±0.3 |
| c-Src N45S | 19.0±0.6 | -6.44±0.02 | 0.19±0.04 | -14.1±0.2 | 0.1±0.3 |
| c- Src W56L | 72.5±1.4 | -5.65±0.01 | 0.98±0.03 | -11.8±0.1 | 2.4±0.2 |
| c-Src H59R | 11.0±0.2 | -6.76±0.01 | -0.13±0.03 | -14.3±0.2 | -0.1±0.3 |
| c-Src R73E | 9.3±0.5 | -6.87±0.03 | -0.24±0.04 | -14.3±0.2 | -0.1±0.3 |
| c-Src R73K | 11.3±0.4 | -6.75±0.02 | -0.12±0.04 | -13.9±0.1 | 0.3±0.2 |
| c- Src R73Q | 11.4±0.3 | -6.74±0.02 | -0.11±0.04 | -14.6±0.1 | -0.4±0.2 |
| c- Src I77V | 15.5±0.3 | -6.32±0.01 | 0.31±0.03 | -13.7±0.1 | 0.5±0.2 |
