# Supplementary material for: From Binding-Induced Dynamic Effects in SH3 Structures to Evolutionary Conserved Sectors
Source: PLoS Comput Biol. 2016 May 23;12(5):e1004938. doi: 10.1371/journal.pcbi.1004938 (PMC4877006; doi:10.1371/journal.pcbi.1004938)
Supplement: S4 Table — Folding parameters of c-Src SH3 domains and diverse mutants, obtained from a multiple curve fitting of all DSC experiments, considering common CpN and CpU functions for Src wt and all mutants analysed. (DOC) [file pcbi.1004938.s009.doc]

| **Protein** | **T_m_ (^o^C)** | **∆H_m_**  **(kcal/mol)** | **∆G_U_ (25 ºC)**  **(kcal/mol)** | **∆∆G_U_ (25 ºC)**  **(kcal/mol)** | **∆H_U_ (25 ºC)**  **(kcal/mol)** | **∆∆H_U_ (25 ºC)**  **(kcal/mol)** |
| --- | --- | --- | --- | --- | --- | --- |
| c-Src wt | 75.53±0.04 | 49.6±0.1 | 4.9±0.2 | 0 | 16.2±3.1 | 0 |
| c-Src F18L | 69.61±0.04 | 46.9±0.1 | 4.2±0.2 | -0.69±0.08 | 16.3±2.7 | 0.2±0.6 |
| c-Src F18W | 77.91±0.02 | 58.2±0.1 | 6.3±0.3 | 1.44±0.05 | 23.8±3.2 | 7.6±0.4 |
| c-Src S26A | 81.30±0.01 | 59.3±0.1 | 6.8±0.3 | 1.88±0.08 | 23.5±3.1 | 7.3±0.6 |
| c-Src L32V | 68.51±0.05 | 43.1±0.1 | 3.7±0.2 | -1.22±0.09 | 13.1±2.7 | -3.0±0.7 |
| c-Src F34I | 74.06±0.04 | 47.5±0.1 | 4.5±0.2 | -0.37±0.05 | 14.8±3.0 | -1.4±0.4 |
| c-Src L40I | 71.48±0.03 | 48.8±0.1 | 4.5±0.2 | -0.33±0.07 | 17.3±2.8 | 1.2±0.5 |
| c-Src L40V | 64.90±0.06 | 41.7±0.1 | 3.3±0.2 | -1.56±0.14 | 13.6±2.7 | -2.6±1.2 |
| c-Src N45S | 77.06±0.02 | 54.2±0.1 | 5.7±0.3 | 0.78±0.04 | 20.2±3.1 | 4.0±0.3 |
| c- Src W56L | 72.61±0.03 | 46.3±0.1 | 4.3±0.2 | -0.61±0.05 | 14.3±2.8 | -1.9±0.4 |
| c-Src H59R | 72.42±0.05 | 52.2±0.2 | 5.1±0.22 | 0.19±0.07 | 20.3±2.9 | 4.1±0.5 |
| c-Src R73E | 68.79±0.02 | 46.8±0.1 | 4.1±0.2 | -0.74±0.08 | 16.7±2.6 | 0.5±0.6 |
| c-Src R73K | 77.51±0.04 | 51.7±0.1 | 5.3±0.3 | 0.43±0.05 | 17.4±3.2 | 1.2±0.4 |
| c- Src R73Q | 73.34±0.04 | 50.5±0.1 | 4.9±0.2 | 0.01±0.05 | 18.1±2.9 | 2.0±0.4 |
| c- Src I77V | 75.57±0.03 | 54.6±0.1 | 5.6±0.2 | 0.74±0.04 | 21.2±3.0 | 5.0±0.3 |
