# Supplementary material for: From Binding-Induced Dynamic Effects in SH3 Structures to Evolutionary Conserved Sectors
Source: PLoS Comput Biol. 2016 May 23;12(5):e1004938. doi: 10.1371/journal.pcbi.1004938 (PMC4877006; doi:10.1371/journal.pcbi.1004938)
Supplement: S5 Table — Experiments were repeated four times. Both the raw results, the transformations relative to the WT and the averages (and standard deviations) are reported. (DOCX) [file pcbi.1004938.s010.docx]

|  | Raw |  |  |  | Fold Change |  |  |  |  |  |
| --- | --- | --- | --- | --- | --- | --- | --- | --- | --- | --- |
| **Experiment** | **215** | **216** | **217** | **219** | **215** | **216** | **217** | **219** | **Average** | **Stdev** |
| Y416F | 0,04 | 0,14 | 0,05 | 0,04 | 0,04 | 0,30 | 0,06 | 0,04 | 0,11 | 0,12 |
| WT | 0,94 | 0,47 | 0,87 | 0,98 | 1,00 | 1,00 | 1,00 | 1,00 | 1,00 | 0,00 |
| D99N | 1,25 | 1,10 | 0,91 | 1,46 | 1,34 | 2,31 | 1,04 | 1,49 | 1,54 | 0,54 |
| L100V | 1,18 | 1,00 | 0,96 | 1,11 | 1,26 | 2,12 | 1,10 | 1,13 | 1,40 | 0,48 |
| F102A | 0,73 | 0,47 | 0,75 | 0,71 | 0,78 | 0,99 | 0,86 | 0,73 | 0,84 | 0,12 |
| W119L | 0,60 | 0,45 | 0,52 | 0,62 | 0,64 | 0,94 | 0,60 | 0,63 | 0,70 | 0,16 |
| Y524F | 1,18 | 2,06 | 2,38 | 4,91 | 1,26 | 4,34 | 2,73 | 4,99 | 3,33 | 1,67 |
